# Effects of psychosis-associated genetic markers on brain volumetry: a systematic review of replicated findings and an independent validation

**DOI:** 10.1017/S0033291722002896

**Published:** 2022-12

**Authors:** Nuno Vouga Ribeiro, Vânia Tavares, Elvira Bramon, Timothea Toulopoulou, Isabel Valli, Sukhi Shergill, Robin Murray, Diana Prata

**Affiliations:** 1 Faculdade de Medicina, Universidade de Lisboa, Lisboa, Portugal; 2 Instituto de Biofísica e Engenharia Biomédica, Faculdade de Ciências, Universidade de Lisboa, Lisboa, Portugal; 3 Division of Psychiatry, University College London, London, UK; 4 Department of Psychosis Studies, Institute of Psychiatry, Psychology & Neuroscience, King’ College London, London, UK; 5 Institute of Cognitive Neuroscience, University College London, London, UK; 6 Department of Psychology & National Magnetic Resonance Research Center (UMRAM), Aysel Sabuncu Brain Research Centre (ASBAM), Bilkent University, Ankara, Turkey; 7 Institut d'Investigacions Biomèdiques August Pi i Sunyer (IDIBAPS), University of Barcelona, Barcelona, Spain; 8 Department of Old Age Psychiatry, Institute of Psychiatry, Psychology & Neuroscience, King's College London, London, UK

**Keywords:** Brain structure, candidate genes, GWAS, imaging genetics, MRI

## Abstract

**Background:**

Given psychotic illnesses' high heritability and associations with brain structure, numerous neuroimaging-genetics findings have been reported in the last two decades. However, few findings have been replicated. In the present independent sample we aimed to replicate any psychosis-implicated SNPs (single nucleotide polymorphisms), which had previously shown at least two main effects on brain volume.

**Methods:**

A systematic review for SNPs showing a replicated effect on brain volume yielded 25 studies implicating seven SNPs in five genes. Their effect was then tested in 113 subjects with either schizophrenia, bipolar disorder, ‘at risk mental state’ or healthy state, for whole-brain and region-of-interest (ROI) associations with grey and white matter volume changes, using voxel-based morphometry.

**Results:**

We found FWER-corrected (Family-wise error rate) (i.e. statistically significant) associations of: (1) *CACNA1C*-rs769087-A with larger bilateral hippocampus and thalamus white matter, across the whole brain; and (2) *CACNA1C*-rs769087-A with larger superior frontal gyrus, as ROI. Higher replication concordance with existing literature was found, in decreasing order, for: (1) *CACNA1C*-rs769087-A, with larger dorsolateral-prefrontal/superior frontal gyrus and hippocampi (both with anatomical and directional concordance); (2) *ZNF804A*-rs11681373-A, with smaller angular gyrus grey matter and rectus gyri white matter (both with anatomical and directional concordance); and (3) *BDNF-*rs6265-T with superior frontal and middle cingulate gyri volume change (with anatomical and allelic concordance).

**Conclusions:**

Most literature findings were not herein replicated. Nevertheless, high degree/likelihood of replication was found for two genome-wide association studies- and one candidate-implicated SNPs, supporting their involvement in psychosis and brain structure.

## Introduction

Schizophrenia is associated with structural brain abnormalities such as smaller intracranial, total brain and total grey matter (GM) volumes (Chung & Cannon, 2015; Kuo & Pogue-Geile, 2019), usually accompanied by an enlargement of the ventricles [for a review see (Chung & Cannon, 2015; Kuo & Pogue-Geile, 2019; Shepherd, Laurens, Matheson, Carr, & Green, 2012); for a meta-analysis see (Kuo & Pogue-Geile, 2019)] Particularly affected are the prefrontal, medial and superior temporal cortices and the hippocampus. Temporo-cortical loss, in particular in the superior temporal gyrus (important for auditory-language processing), has been associated with psychotic symptoms (i.e. hallucinations, delusions and formal thought disorder) (Jung, Lee, Bang, & Lee, 2019; Kasai et al., 2003). Moreover, such loss appears to worsen with psychosis progression (Ho et al., 2003; Kasai et al., 2003; Nakamura et al., 2007). Besides schizophrenia (Kuo & Pogue-Geile, 2019), hippocampal volume loss has also been well established in bipolar disorder with psychotic symptoms (Haukvik, Tamnes, Söderman, & Agartz, 2018). In addition, to avoid the potential confounder of antipsychotic medication (Navari & Dazzan, 2009; Voineskos et al., 2020), research in individuals with an ‘At Risk Mental State’ ARMS, usually untreated pharmacologically, has also been conducted (Andreou & Borgwardt, 2020). This prodromal phase of subthreshold psychotic symptoms (Fusar-Poli, Carpenter, Woods, & McGlashan, 2014) is associated with a risk of transition to the schizophrenia of about 16%, and to bipolar disorder with psychotic features of about 2%, within a period of 2.35 years (Fusar-Poli et al., 2013). In ARMS subjects volumetric reductions have been reported in the hippocampus, parahippocampus, cingulate, medial and lateral frontal, and parietal cortices (Bois, Whalley, McIntosh, & Lawrie, 2015; Fusar-Poli et al., 2011), particularly in those who transitioned to psychosis (Borgwardt et al., 2008; Dazzan et al., 2012; Pantelis et al., 2003; Takahashi et al., 2009). White matter (WM) volume has been studied to a lesser extent [compared to GM's], but reductions have also been reported in schizophrenia in the prefrontal cortex (Breier et al., 1992; Haijma et al., 2013; Hulshoff Pol et al., 2002; Suzuki et al., 2002; Wagner et al., 2013), the internal capsule and other regions (Suzuki et al., 2002; Zhou et al., 2003). Furthermore, WM reductions in these structures are also suggested to underlie cognitive impairments seen in patients (Nagy, Westerberg, & Klingberg, 2004; Suzuki et al., 2002; Wagner et al., 2013; Zhou et al., 2003), and to worsen with illness progression, starting in the prodromal phase (Witthaus et al., 2008) . For instance, individuals with an ARMS who later transitioned to psychosis, *vs.* those who did not, have shown: (a) reduced WM volume in the right superior temporal lobe, when compared with healthy controls (Witthaus et al., 2008); (b) larger WM volume in the left frontal lobe (Walterfang et al., 2008); and (c) WM volume reduction in the left fronto-occipital fasciculus (Walterfang et al., 2008).

The aetiology of schizophrenia and bipolar disorder has a strong genetic component. Heritability has been reported as high as 80% for schizophrenia, and 93% for bipolar disorder (Kieseppä, Partonen, Haukka, Kaprio, & Lönnqvist, 2004; Sullivan, Kendler, & Neale, 2003), with a substantial overlap between the two (Avissar & Schreiber, 2002; Knight et al., 2009; Lichtenstein et al., 2009). The identification of the specific risk variants has been harder but pushed forward by genome-wide association studies (GWAS) in the last decade, largely replacing the earlier candidate-gene approach. Neuroimaging and molecular genetics research have combined forces in a neuroimaging genetics approach, which could go a step further by building on both types of findings and testing the effect of genetic variations that have been previously associated with psychosis, on brain functional or structural (intermediate) phenotypes, in an attempt to clarify pathophysiological mechanisms, and even create new biomarkers. We and others have frequently followed this endeavour, supporting evidence for some psychosis genes' involvement in brain function (Costafreda et al., 2009; Dahoun et al., 2018; Gurung & Prata, 2015; Mechelli et al., 2008, 2012, 2009; Nosarti et al., 2011; Papagni et al., 2011; Pauli et al., 2013; Pettersson-Yeo et al., 2013; Prata et al., 2008, 2011; Prata et al., 2009b, 2009c, 2009d; Prata et al., 2012; Radua et al., 2013; Ranlund et al., 2018; Tecelão et al., 2018, 2019) and structure (Dutt et al., 2009; Mallas et al., 2016; Mallas et al., 2017; Prata et al., 2013; Radua et al., 2013; Simões et al., 2020). However, such findings have been variable and their reproducibility should be tested, as should that of every statistical finding using potentially underpowered, under-representative or phenotypically heterogeneous samples, which is not uncommon in psychiatry. Insufficient replication attempts of many of these findings persist (Bogdan et al., 2017), as well as inconsistencies on the anatomical or directional effects of risk genes on brain structure (Hashimoto et al., 2015) and false positives coming from insufficient correction for multiple comparisons (Gurung & Prata, 2015).

In the present study, we aimed to validate previously published psychosis-implicated imaging genetics findings in an independent imaging genetics sample including schizophrenia, bipolar disorder, ARMS and healthy individuals, using a whole-brain and a region-of-interest (ROI) approach. To further reduce false positives, we focused on testing only the effect of those genetic variants which were previously found to have a statistically significant effect at least twice (in two independent samples/studies) on any regional GM or WM volume. For completeness, we also discuss consistency in findings between previous studies and between such studies and our own, regarding anatomical location and allelic direction.

## Methods and materials

### Independent sample data

#### Sampling

We examined a total sample of 113 subjects, including 14 patients with schizophrenia and 21 with bipolar disorder, 34 with an ARMS (9 who later transitioned to psychosis, ARMS-T, and 25 who did not, ARMS-NT), and 44 healthy volunteers (demographics in Table S20). Patients and healthy volunteers were in a stable clinical state when participating and were recruited from the South London and Maudsley (SLaM) trust as part of the Genetics and Psychosis (GAP) study (Di Forti et al., 2015). Diagnosis, according to the DSM-IV (American Psychiatric Association, 2000), was ascertained by an experienced psychiatrist using a structured diagnostic interview with instruments detailed elsewhere (Prata et al., 2009a, 2009b, 2009c). ARMS individuals were recruited at the OASIS service (Broome et al., 2005), and assessed using the CAARMS (Phillips, Yung, & McGorry, 2000). The study was approved by the NHS South East London Research Ethics Committee (Project GAP; Ref. 047/04), consistent with the Helsinki Declaration of 1975 (as revised in 2008) and all subjects gave written informed consent. Further recruitment details are in online Supplementary Material.

#### Genotyping and imputation

Genotyping procedures have been described in previous publications (Bramon et al., 2014; Vassos et al., 2017) and in this article's online Supplementary Material.

#### Image acquisition and pre-processing

Structural magnetic resonance imaging (MRI) scans were acquired with two different scanners using eight different protocols. T1-weighted images were free of artefacts (assessed through visual inspection) and processed with CAT12 [v1092 (Jena, Germany) (Gaser, Dahnke, Kurth, & Luders, n.d.)], a SPM12 add-on (v6909) (London, UK) (Penny, Friston, Ashburner, Kiebel, & Nichols, 2006) using default settings and MATLAB (9.1) (Natick, Massachusetts) (*MATLAB*, 2016). Firstly, bias field inhomogeneity correction was performed. Secondly, images were segmented into GM, WM, and cerebrospinal fluid. Thirdly, images were spatially normalized to a template from the IXI dataset (Biomedical Image Analysis Group, Imperial College London (2015). Information eXtraction from Images (IXI) dataset) using the DARTEL (Ashburner, 2007) algorithm. Finally, Jacobian scaled (‘modulated’) warped tissue maps were then created for both GM and WM, and the resultant images were then smoothed with an 8 × 8 × 8 mm Gaussian kernel. Moreover, the total intracranial volume (TIV) was computed by summing all voxels classified as GM or WM or as cerebrospinal fluid. Further details are in online Supplementary Material.

### Systematic review

#### SNP selection

**Fig. 1.**
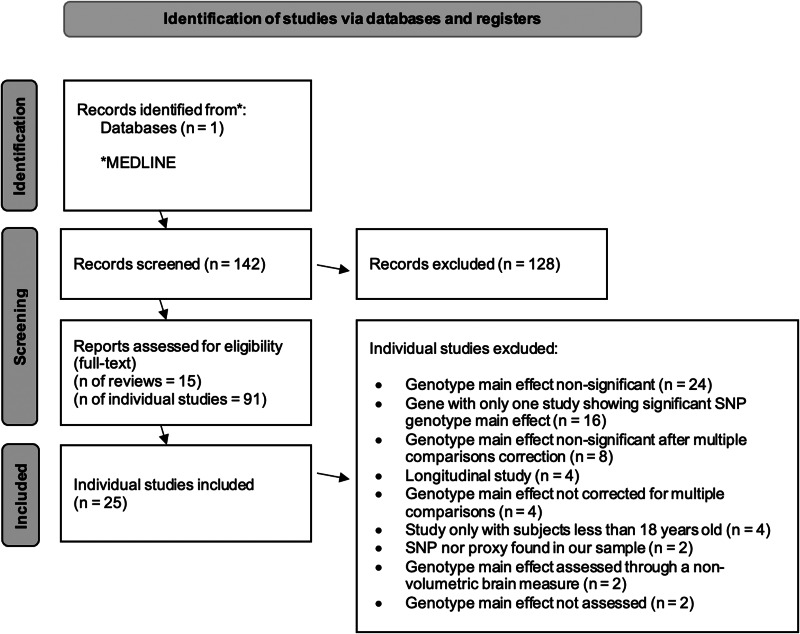
PRISMA flow diagram for systematic review studies selection.

A systematic bibliographic search was performed (by author NVR) to select the SNPs whose effect on brain structure was to be validated in our imaging genetics sample. The MEDLINE database was searched (in November 2018) for reviews of studies investigating the effect of SNPs on brain volumes of either patients with psychotic disorders (schizophrenia or bipolar disorder) or healthy individuals. The following broad search syntax was used: [Gene AND neuroimaging] AND [Bipolar and Related Disorders OR Schizophrenia Spectrum and Other Psychotic Disorders], with a filter for ‘Review’ and no language restriction. The search resulted in 142 papers. Of this set, abstracts were screened for reviews aggregating studies corresponding with our study objectives. After one review was excluded due to language reasons (Japanese), 15 reviews of interest remained – 6 systematic, (Duff, Macritchie, Moorhead, Lawrie, & Blackwood, 2013; Gurung & Prata, 2015; Kurnianingsih et al., 2011; Najjar & Pearlman, 2015; Pereira et al., 2017; Sun et al., 2015) 9 non-systematic (Chang, Xiao, & Li, 2017; Fineberg & Ellman, 2014; Martin, Robinson, Dzafic, Reutens, & Mowry, 2014; Meyer-Lindenberg, 2010; Mostaid et al., 2016; Notaras, Hill, & van den Buuse, 2015; Redpath et al., 2013; van Haren, Bakker, & Kahn, 2008; Voineskos, 2015) – and their full-text was analysed. Further screening through the abstracts of the individual studies included in these reviews and removal of duplicates resulted in 90 studies of interest, potentially fulfilling all our SNP inclusion criteria (detailed below). After full-text analysis for complete compliance with those criteria, only 25 of these studies were selected (see Fig. 1 for a flow diagram of study selection, Tables 1, 2, S21 and S22 for included studies, and Table S19 for further information on the 66 excluded studies).

**Table 1.** BDNF and CACNA1C: primary studies of its SNPs of interest and their corresponding effects on brain volume; in comparison with the present study's findings

Primary study				Present study [113 (HC: 44; SZ: 14; BD: 21; ARMS: 34); White European & North American]			
Gene SNP	Authors Year	Genotype effect^a^	Whole-brain region	Proxy SNP; allele (*r*^2^)	Statistical model^c^	Whole-brain^b^grey natter (peak Z)	Whole-brain white matter (peak Z)
*BDNF* rs6265	Ho et al. (2006)	T-car. < CC	Temporal and Occipital lobes, L Inf Temporal, L Sup Frontal, L Lat Occipital, L Parahippocampal, L Supramarginal (GM)	rs4923457; T = T, C = A; (0.80)	T-car. *vs.* AA	L Postcentral (*Z* = 3.38, T-car. >AA)	L Inf Parietal (*Z* = 4.11, T-car.>AA)
	Montag et al. (2009)		Bilateral parahippocampal, L Ant hippocampus, R Amygdala (GM)				
	Chepenik et al. (2009)		Bilateral Hippocampus				
	Nemoto et al. (2006)		L Parahippocampal, Bilateral heads of caudate nucleus (GM)				
	Pezawas et al. (2004)		Bilateral Hippocampus, Bilateral DLPFC (GM)				
	Yang et al. (2012)		Bilateral: Sup and Mid Frontal, Mid Temporal, Sup Parietal, Insula; L: Orb Frontal, Lat Occipital (Cortical thickness)				
	Szeszko et al. (2005)	TC < CC	Bilateral Hippocampus		TA *vs.* AA	L Cuneus (*Z* = 3.94, TA<AA) L Mid Cingulate (*Z* = 3.44, TA > AA) R Sup Frontal (*Z* = 3.54, TA > AA)	R Sup Longitudinal fasciculus (*Z* = 3.69, TA<AA)
	Bueller et al. (2006)		Bilateral Hippocampus				
	Yang et al. (2012)		L Mid Cingulate, L Insula (Cortical thickness)				
	Matsuo et al. (2009)		Bilateral DLPFC, Bilateral Ant Cingulate, L Hippocampus (GM)				
*CACNA1C* rs1006737	Perrier et al. (2011)	A-car. > GG	R Amygdala, R Hippocampus (GM)	rs769087; A~A, G~G; (1.0)	A-car. *vs.* GG	**ROI-analysis: R Sup Frontal (*Z* = 3.42, A-car.>GG; *η*_p_^2^ = 0.049**^d^**) (GM)**	R Inf Temp (*Z* = 3.69, A-car.<GG) L Orb Mid Front (A-car.<GG)
	Wang et al. (2011)		Bilateral ventral, rostral and DLPFC, Ant Cingulate and Temporal cortices (GM)				
	Wolf (2014)		Bilateral Amygdala (GM)				
	Franke et al. (2010)	AA < AG < GG	Total volume (GM)		AA *vs.* AG *vs.* GG	R Calcarine (*Z* = 3.57, AA>AG) L Fusiform (*Z* = 3.30, AA<AG)	**L Hippocampus (*Z* = 4.66, FWER *p* = .026^c^, AA>AG; AA>GG; *η*_p_^2^ = 0.302**^d^**) L + R Thalamus, R Hippocampus (*Z* = 4.58, FWER *p* = .036^c^, AA>AG;AA>GG; *η*_p_^2^ = 0.264**^d^**)** R Cuneus (*Z* = 4.09, AA>AG) L Mid Temporal, R Fusiform, L Postcentral, R Inf Occipital, L Mid Frontal, L Cingulate (AA>AG) R Caudate nucleus head, L Frontal Lobe Sub-Gyral, L Sup Occipital (AA<AG; AA<GG)

Ant, Anterior; ARMS, At Risk Mental State; BD, Bipolar disorder; car., Carriers; DLPFC, Dorsolateral prefrontal cortex; GM, grey matter; HC, Healthy controls; Inf, Inferior; L, Left; Lat, Lateral; Med, Medial; Mid, Middle; Orb, Orbital; PFC, Prefrontal cortex; Post, Posterior; R, Right; ROI, Region of interest; Sup, Superior; SZ, Schizophrenia; VL, Ventrolateral; WM, White matter.

^a^ risk genotype always on the left; ^b^ also including results from region-of-interest (ROI) analyses, when specified;^c^ significant after FWER-correction, but only for the peak of effect (both peaks with a cluster size of six voxels on the genotype main effect *F*-test); ^d^ extracted through volume of the peak voxel.

Coordinates of effects in tables S1 and S2.

**Table 2.** ZNF804A: primary studies of its SNPs of interest and their corresponding effects on brain volume; in comparison with the present study's findings

Primary study				Present study [113 (HC: 44; SZ: 14; BD: 21; ARMS: 34); White European & North American]			
Gene SNP	Authors Year	Genotype effect^c^	Whole-brain region	Proxy SNP; allele (*r*^2^)	Statistical model^c^	Whole-brain grey matter (peak Z)	Whole-brain white matter (peak Z)
*ZNF804A* rs1344706	Voineskos et al. (2011)	AA < C-car.	R Ant Cingulate, L Post Cingulate, L Sup Temporal (Cortical thickness)	rs11681373; A~A, C~G; (1.0)	AA *vs.* G-car	R Angular (*Z* = 3.74 AG>GG) L Calcarine, L Inf Occipital, R Precentral (AG<GG)	R Inf Occipital (*Z* = 4.00, AA<G-car.) L Postcentral, L Precuneus, R Sub-lobar Extra-Nuclear, L Rectus (AA<G-car.)
	Donohoe et al. (2011)	AA > C-car.	Bilateral Hippocampus and Amygdala				
	Lencz et al. (2010)	AA^a^ > C-car.	Total volume (WM)				
		AA < C-car.	L Angular gyrus, R Parahippocampal gyrus, R Post Cingulate, R Inf Temporal gyrus, Bilateral rectus gyrus, R Angular gyrus, R Cerebellum (posterior uvula), L Inf Frontal gyrus (Broca's), R Cerebellum (posterior tonsil) (GM)				
	Schultz et al. (2014)	AA > C-car.	(Cortical thickness) L: DLPFC, VL PFC, Broca's area, Med Sup Frontal, Orbitofrontal, Sup Temporal, Mid and Inf Temporal, Ant Med Temporal, Inf Parietal; R: DLPFC, Inf Frontal, Sup Temporal, Mid and Inf Temporal, Inf Parietal, Lat Occipital, Med Sup Frontal, Parahippocampal				
	Wassink et al. (2012)	A-car. > CC AA > AC > CC	Frontal lobe (WM)		A-car. *vs.* GG^d^; AA *vs.* AG *vs.* GG		R Inf Occipital (*Z* = 4.30, AA<GG); L Orb Sup Frontal (*Z* = 3.51 AA<AG)
	Wei et al. (2012)	A-car. > CC^b^	L and R Hippocampus (WM density)		AA *vs.* AG *vs.* GG		

Ant, Anterior; ARMS, At Risk Mental State; BD, Bipolar disorder; car., Carriers; DLPFC, Dorsolateral prefrontal cortex; GM, grey matter; HC, Healthy controls; Inf, Inferior; L, Left; Lat, Lateral; Med, Medial; Mid, Middle; Orb, Orbital; PFC, Prefrontal cortex; Post, Posterior; R, Right; ROI, Region of interest; Sup, Superior; SZ, Schizophrenia; VL, Ventrolateral; WM, White matter.

Originally reported as ^a^ TT *vs.* C-car. in Lencz et al., 2010, and ^b^ T-car. *vs.* GG in Wei et al., 2012; ^c^ risk genotype always on the left; ^d^ excluded due to ‘scan protocol’ being found to be a true confounder variable.

Coordinates of effects in tables S1 and S2.

The SNP inclusion criteria, all necessarily met by the selected individual studies, were: (a) the SNP had to be present within a gene previously associated with a psychotic disorder (i.e. schizophrenia or bipolar disorder – evidence of association is described in this article's Discussion section); (b) the SNP risk allele had to show a statistically significant main effect (surviving multiple comparisons correction) on any brain region volume, whether it be in healthy subjects or patients with psychotic disorders; (c) this effect had to be assessed through structural MRI, with at least 1.5 T of strength, and the measures of brain structure were either total or regional GM or WM or ventricle volumes, total or regional cortical thickness or total or regional GM or WM density; and (d) each SNP selected had to fulfil all these criteria in at least two different individual studies, using independent samples. SNPs were excluded if the studies referred to: (a) Diffusion Tensor Imaging measurements, (b) participants younger than 18 years old, or with any childhood-onset psychotic disorder, (c) studying the effect of *COMT* rs4680 polymorphism – which codes for Val158Met amino acid change – on brain structure, given the already extensive previous research and replication attempts in the literature (Ira, Zanoni, Ruggeri, Dazzan, & Tosato, 2013), (d) assessing only the effect of genetic markers other than SNPs and (e) studying longitudinal effects. Doubts regarding the inclusion of individual studies were resolved by authors VT and DP.

Eleven SNPs were then selected, within five different genes. Data from the individual studies of interest (herein called ‘primary studies’) (Bueller et al., 2006; Cannon et al., 2012; Chepenik et al., 2009; Donohoe et al., 2011; Franke et al., 2010; Ho et al., 2006; Kähler et al., 2012; Lencz et al., 2010; Mata et al., 2010, 2009; Matsuo et al., 2009; McIntosh et al., 2007; Montag, Weber, Fliessbach, Elger, & Reuter, 2009; Nemoto et al., 2006; Perrier et al., 2011; Pezawas et al., 2004; Schultz et al., 2014; Szeszko et al., 2005; Tosato et al., 2012; Voineskos et al., 2011; Wang et al., 2011; Wassink et al., 2012; Wei et al., 2012; Wolf et al., 2014; Yang et al., 2012) was collected, namely: the SNP and gene examined, the source study, the significant (according to our inclusion criteria) morphological main effects, the study population and provided coordinates of ‘peak effects’ with the corresponding brain structure/measure (to perform ROI-analyses) (Tables 1, 2, S21 and S22).

### SNPs retrieved through systematic review and analysed in our independent sample

#### SNPs match to our sample

SNP selection in our sample was done using the PLINK software (version 1.9) (Chang et al., 2015). The ARMS and GAP samples were first modelled to fit the typical population from which the SNPs of interest were obtained (mostly ‘White Europeans/North Americans’). We validated ancestry through a population stratification analysis using principal component analysis (Figure S7). The search for the SNPs was done using their genomic location. We sought to select appropriate proxies, among those we genotyped, for the remaining unmatched SNPs whenever there was no exact match (criteria and workflow for SNP proxies selection are to be found in the online Supplementary Material). This resulted in a total of seven testable SNPs (in five different genes). Through LD-link, the risk alleles of these proxy SNPs were matched with the risk alleles of the SNPs featured in the primary studies (Tables 1, 2 and S22).

#### Neuroimaging genetics analysis

The effect each SNP's genotype had on GM and WM volume was tested using a whole-brain analysis and a ROI analysis. For the whole-brain analysis, we have run an analysis of variance with the voxel-based GM and WM volume maps as dependent variables (i.e. one statistical model per brain tissue type) and the subjects' SNP genotype as an independent variable (i.e. one statistical model per SNP). Genotypes were coded according to either a dominant, recessive or additive model (as detailed in online Supplementary Material). For each SNP, the classification of alleles as ‘risk/non-risk’ and the choice of model was made entirely based on the allele classification and model used by the primary studies from which the SNPs were selected. When different models had been used for each SNP, all were tested. For completeness, for each SNP model, we tested for a ‘negative’ (decreasing volume) and a ‘positive’ (increasing volume) effect of genotype on brain volume in whole-brain analyses. Furthermore, age, sex, handedness, diagnosis (i.e. schizophrenia, bipolar disorder, ARMS-T, ARMS-NT, and healthy controls), TIV, and scan protocol were inserted in the statistical models as covariates of no interest. Analyses where these covariates happened to be simultaneously correlated with our genotype variable and brain phenotype variables were excluded, given the high risk of producing confounded results, namely: the comparisons relating to *NRG1 rs35753505* and *rs4733264* and the *ZNF804A rs11681373*1 A carriers *vs.* GG comparison.

To achieve a more precise validation (closer to a *de facto* replication) of previously reported SNP's genotype effects on brain volume, we conducted ROI analyses using the brain region/measure, direction of effect and genotype comparison reported by the corresponding primary study. Such analyses were conducted using peaks of genotype effect reported by the primary studies, from which individual masks (either spherical or regional) were built, using the WFU pickatlas SPM12 add-on (Maldjian, Laurienti, Kraft, & Burdette, 2003). More details can be found in the online Supplementary Material.

All whole-brain and ROI statistical analyses were defined using a full factorial design using SPM12 and its add-on CAT12. The effect of each SNP's genotype on GM or WM volume was only considered to be statistically significant (with no cluster size cut-off): (a) at a whole-brain level, at *p*-value < 0.05 after correction for multiple testing (i.e. for the number of voxels) using a voxel-level Family-wise Error Rate (FWER); and (b) on an ROI level, at *p*-value <0.001 uncorrected. All other effects with an uncorrected *p*-value < 0.001, and exceeding a cluster size cut-off of 25 voxels, are reported as ‘trends’. In all analyses, significant and trend results were mapped anatomically using the ‘xjview’ toolbox (v10.0) (https://www.alivelearn.net/xjview) and, in the case of large resulting clusters (>200 voxels), further confirmed manually through printed book atlases.

Since our study has increased protection against false positives given it aimed at the validation of previous (at least partially) replicated findings of SNP effects, we have not corrected our analyses for the number of SNPs tested (*N* = 7) nor modalities (*N* = 2, WM and GM) nor ROIs per SNP tested (different for each SNP, given it was based on previous evidence for each SNP; an average of eight ROIs per SNP were performed).

To assess how much of the interindividual variance in regional brain volume was explained by the genetic variation, we used for each significant result the *η*_p_^2^ (partial eta squared) measure of effect size, calculated in SPSS, after extracting the subjects' volume measure at the voxel of peak difference from SPM (details in online Supplemental).

All mentions of genotype comparisons, either from the primary studies or the present study, both in the main and supplementary manuscript, tables and figures, are stated as ‘high-risk allele load *vs.* low-risk allele load’.

## Results

### Concordance between primary studies

Heterogeneity was found between the primary studies, on sample diagnostic composition and ancestry, in a variable degree (Table S21), as well as regarding genotypic or imaging procedures or adjustment for covariates of no interest.

Still, regarding the primary studies results, an overall low concordance in terms of the anatomical location of effects per SNP was found, given that only those for *BDNF*-rs6265, *ZNF804A*-rs1344706 and *CACNA1C*-rs1006737 – among seven SNPs (in five different genes) – showed some anatomical overlap between at least two selected studies (Tables 1 and 2). In the case of *BDNF*-rs6265-T, 6 of those studies reported a reduction of hippocampal volume, 3 a reduction of the parahippocampal gyrus, 3 a reduction of the bilateral dorsolateral prefrontal GM, and 2 a reduction of the cingulate cortex volumes. For *ZNF804A*-rs1344706-A, 2 reported an effect on Broca's area, inferior temporal gyrus and parahippocampal gyrus, although for all those areas one primary study showed a GM reduction, while the other showed an increased cortical thickness. For *CACNA1C*-rs1006737-A, 2 reported an increase of amygdala volume.

Concordance in terms of the genotype effect on brain matter type was also higher for the same aforementioned three SNPs: *BDNF*-rs6265-T was associated with a general decrease of GM volume; *ZNF804A-*rs1344706-A was associated with increased WM volume and decreased GM volume (although with inconsistent findings regarding cortical thickness); and *CACNA1C*-rs1006737-A was associated with increased GM volume in three studies (although a fourth one reported an inverse association, on total GM volume).

#### Primary and present studies concordance in whole-brain findings

In order to simplify the interpretation of the significance and extension of the overlap between results of the primary and present studies for each SNP on a putative functional level, a criterion of ‘replication concordance’ was used, composed of five different parameters, here presented in decreasing order of importance: anatomy (‘A’; an effect on brain volume effect is found for the same brain region, between the primary and present studies), direction (‘Dir’; the effect is found to either ‘increase’ or ‘decrease’ a particular brain volume), brain matter (‘Bm’; the effect is found on the same brain matter type, either GM or WM), laterality (‘Lat’; the effect is found, for a particular brain region, on the same brain hemisphere; with ‘1/2 Lat’ meaning only concordance in one brain hemisphere was found) and genotype (‘Gen’; the effect is found for the same genotype effect model used in the primary and present study). This nomenclature is used in the following manuscript sections and Table 3.

**Table 3.** Most approximate replication concordance, of main effects of SNP genotypes on grey or white matter volume, between our study and primary studies findings from the systematic review performed

Analysis	Gene SNP (Proxy)	Effect in primary study sample (risk genotype on the left)	Effect in present study sample (risk genotype on the left)	Primary-present study replication concordance^a^^,^^b^				
				Anatomical	Directional	Brain matter	Laterality	Genotype
Whole-brain (FWER-corrected)	*CACNA1C* rs1006737 (rs769087)	(GM) A-car. > GG, right hippocampus	(WM) AA>AG and AA>GG, L + R hippocampus					
Whole-brain (Present Study Trend)	*ZNF804A* rs1344706 (rs11681373)	(GM) AA < C-car., L + R angular gyrus	(GM) AA < AG, R angular gyrus					
		(GM) AA < C-car., bilateral rectus gyrus	(WM) AA < G-car., L rectus gyrus					
		(WM) A-car.>CC; AA > AC > CC Frontal lobe	(WM) AA<AG, L orbital portion superior frontal					
		(Cortical thickness) AA > C-car., R inferior occipital	(WM) AA< G-car., A-car. < GG, AA < GG, R inferior occipital gyrus					
		(Cortical thickness) AA > C-car., R inferior parietal	(GM) AG > GG, R angular gyrus					
	*BDNF* rs6265 (rs4923457)	(Cortical thickness) TC < CC, L middle cingulate + bilateral anterior cingulate cortex	(GM) TA > AA, L middle cingulate					
		(GM) TC < CC, bilateral DLPFC	(GM) TA > AA, R superior frontal gyrus					
		(GM) T-car. < CC, L and bilateral superior frontal gyri	(GM) TA > AA, R superior frontal gyrus					
		(GM) T-car. < CC, L supramarginal gyrus	(WM) T-car. > AA, L inferior parietal lobe					
		(GM) T-car. < CC, Temporal lobe + L inferior and middle temporal gyrus	(WM) TA < AA, R superior longitudinal fasciculus					
		(GM) T-car. < CC, bilateral parahippocampal gyri	(WM) TA < AA, R superior longitudinal fasciculus					
	*CACNA1C* rs1006737 (rs769087)	(GM) A-car. > GG, anterior cingulate	(WM) AA > AG, L cingulate gyrus					
		(GM) A-car. > GG, temporal cortex	(WM) AA > AG, L middle temporal gyrus					
		(GM) A-car. > GG, bilateral DLPFC	(WM) AA > AG, L middle frontal gyrus					
		(GM) A-car. > GG, rostral PFC	(WM) A-car. < GG, L orbital medial frontal gyrus					
Region-of-interest	*CACNA1C* rs1006737 (rs769087)	(GM) A-car. > GG, L + R DLPFC	(GM) A-car. > GG, R DLPFC, superior frontal gyrus					

BDI, Bipolar disorder type I; car., Carriers; DLPFC, Dorsolateral prefrontal cortex; GM, grey matter; L, Left; PFC, Prefrontal cortex; R, Right; SZ, Schizophrenia; WM, White matter.

a “Replication concordance” being an aggregate measure depicting degree of overlaping effects on a functional level, organized by decreasing order of functional importance: “Anatomical”: anatomical structure; “Direction”: direction of risk genotype effect on volume; “ brain matter”: either grey or white; “Laterality”: brain hemisphere; “Genotype”: genotypic comparison (within each analysis category and SNP, results are organized from top to bottom according to decreasing level of replication concordance). A box fully colored (in grey) means full concordance; a box colored with diagonal lines means close concordance in the case of the “Anatomical” criterion and correspondence just for one of the brain hemispheres in the case of the “Laterality” criterion; ^b^Present study results with no minimal anatomical correspondence with primary results were not included in this table.

**Fig. 2.**
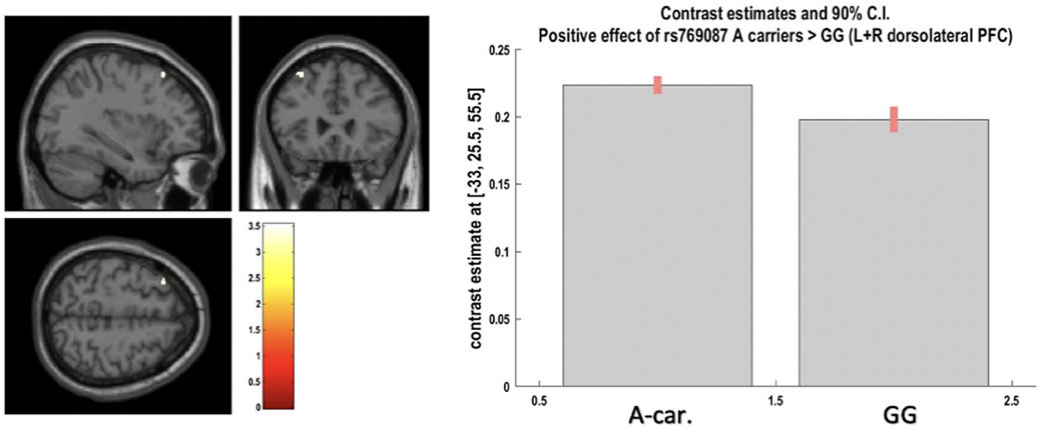
Positive effect of A-carriers v.vs. G homozygotes for CACNA1C rs769087 on left and right dorsolateral prefrontal cortex grey matter at voxel of peak effect (from region-of-interest analysis; at p < 0.05 after FWER correction). The image shows a cluster at p < 0.001 uncorrected, with blue cross-hair lines pointing to the voxel of peak effect.

The present study found the following whole-brain FWER-corrected significant main effect: a rs769087 (*CACNA1C*-rs1006737 proxy), for both AA *vs.* AG and AA *vs.* GG, increased volume on two clusters of WM voxels, respectively located on the left hippocampus (genotype explained 30,2% of the variance in the WM volume variance left unexplained by the other covariates), and left + right thalamus and right hippocampus (genotype explained 26,4% of the otherwise unexplained WM volume variance) (Fig. 2, Tables 1 and 2).

The most approximate anatomical overlap between this FWER-correction significant finding and the ones from the primary studies was found for rs769087 (proxy of *CACNA1C*-rs1006737) on the right hippocampus (A-Dir-1/2Lat; yet the primary study did not test the same genotype model comparison) (Table 3).

The present study also found trend main effects (with uncorrected *p*-value <0.001) for all the SNP's tested, for either GM or WM, on at least one genotype effect model per SNP. Among these trend findings, one effect regarding rs11681373 (*ZNF804A*-rs1344706 proxy) showed high primary and present study concordance (at least A-Dir-Bm-1/2Lat), and two effects regarding rs4923457 (*BDNF*-rs6265 proxy) as well, with at least A-Bm-1/2Lat-Gen (Table 3).

Regarding the already replicated findings between primary studies, a higher primary and present study concordance was found for *BDNF*-rs6265-T association with volume change on superior frontal gyrus GM (A-Bm-1/2Lat-Gen; three primary and the present study reporting it) and cingulate cortex GM (A-Dir-Bm-Lat-Gen; two primary and the present study reporting it). It should be mentioned that, on the present study, all these results were ‘trends’.

### Primary and present studies concordance in present study's ROI analysis findings

**Fig. 3.**
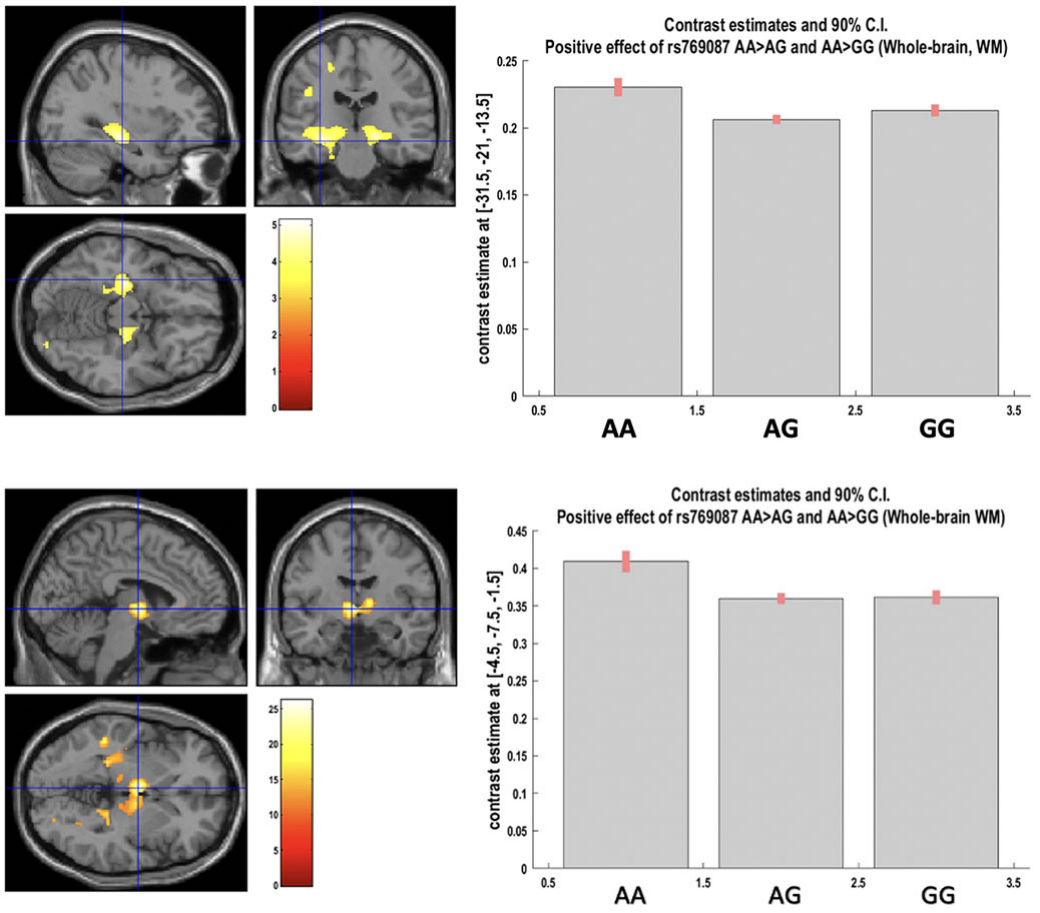
Above: Positive effect of AA v.vs. AG and AA v.vs. GG genotypes for CACNA1C rs769087 on left hippocampus white matter. Below: Positive effect of AA v.vs. AG and AA v.vs. GG genotypes for CACNA1C rs769087 on left and right thalamus white matter (in image), and right hippocampus white matter (not in image). Both images as whole-brain analyses, showing clusters at p < 0.001 uncorrected, and blue cross-hair lines pointing to the voxel of peak effect (at p < 0.05 after FWER correction).

A significant effect was found for rs769087 (*CACNA1C*-rs1006737 proxy) A carriers *vs.* GG, as an increased volume of the bilateral dorsolateral prefrontal cortex (DLPFC) GM, where genotype explained 5% of the region GM volume variance left unexplained by covariates. There was also a high primary and present study concordance for this finding (A-Dir-Bm-1/2Lat-Gen; one primary study) (Fig. 3, Table 1 and 3). All the remaining ROI analyses performed, both coordinates- and region-based (respectively, 23 and 14 analyses – not reported), showed non-significant results.

## Discussion

Both our systematic review findings and the attempt of validation/replication of such results in our independent sample showed mixed results, despite the fact that our study used an overall comparable sample size, and sample diagnostic and ancestry composition (Table S21). In terms of replication broadness, of the roughly 95 different brain regions reported across all primary studies and its SNPs that showed a significant genotype effect after multiple comparisons correction, 19 regions (1 at whole-brain after FWER correction, 17 at whole-brain ‘trend’ and 1 at ROI analysis) had replication concordance with our study (and mostly partial), bearing mention in Table 3.

### Replication concordance per gene

The SNPs with the highest replication concordance correspond roughly to the ones implicated by a higher ratio of ‘studies with significant findings’ to ‘studies with non-significant findings’ (in the literature we have searched; excluding studies not assessing genotype main effects with multiple comparison correction; see Tables 1, 2, S22 and S19): *CACNA1C*-rs1006737 with a ratio of 4, *ZNF804A*-rs1344706 with 3, *NRG1*-rs6994992 with 2, *BDNF*-rs6265 with 1,67 and *NRG1*-rs35753505 with 0,25. Also, a high degree/likelihood of replication was found for the two GWAS- (*ZNF804A* and *CACNA1C*) and one candidate-implicated (*BDNF*) SNPs, which supports their involvement in psychosis and brain structure. Next, we discuss all five genes analysed in the present study, and SNPs, in finer detail, including its association with psychotic disorders.

#### CACNA1C-rs1006737

The *CACNA1C* gene is located on chromosome 12p13.3 and encodes for the a1C subunit of the CaV 1.2 voltage-dependent L-type calcium channel. CaV 1.2 plays an important role in dendritic development, neuronal survival, synaptic plasticity, memory formation, learning and behaviour (Bhat et al., 2012). The intronic polymorphism investigated in this study, rs1006737 A allele, was significantly associated with schizophrenia risk in a large GWAS (Cross-Disorder Group of the Psychiatric Genomics Consortium, 2013), meta-analyses of case–control studies [both in European and Asian populations (Liu, Wu, Xia, Yao, & Wang, 2020; Nie et al., 2015) and in a recent meta-analysis of candidate studies (Zhu et al., 2019)].

*CACNA1C*-rs769087 showed significant results in the present study after FWER correction and at ROI analyses (according to partial eta square, respectively two large effects and one of medium size), both overlapping with the primary studies, in addition to other trend level findings, although without overlap with the primary study. Generally, *CACNA1C*-rs769087-A presented with increased brain volume in some dispersed regions, although in GM in the primary study (consistently) and more in WM in the present study. The association of its risk allele with schizophrenia is substantially supported, a proxy with r^2^ of 1.0 was used in the present study and four primary studies with significant results were considered. However, the primary study sample composition is skewed towards healthy subjects, many findings in the present study for WM were not present in the primary studies and the study that allowed high concordance for DLPFC (A-Bm-1/2Lat-Gen) – Wang *et al*. 2011 (Wang *et al*. 2011) – did not adjust its findings to TIV, which might have significantly confounded its results.

#### BDNF-rs6265

The *BDNF* gene is located on chromosome 11p. It encodes for the BDNF protein, which acts as a survival- and growth-promoting agent on hippocampal and cortical neurons, strengthens excitatory (glutamatergic) synapses, weakens inhibitory (GABAergic) synapses and enhances neurogenesis (Binder & Scharfman, 2004). *BDNF*-rs6265 has been associated with decreased hippocampal volumes in both healthy controls and patients experiencing first-episode psychosis (Pujol et al., 2020).

Studies associating *BDNF*-rs6265 with schizophrenia have shown inconsistent results, even regarding which of its alleles contributes risk: T allele in (Schumacher et al., 2005), C allele in (Neves-Pereira et al., 2005; Rosa et al., 2006). In addition, a meta-analysis of 39 case–control studies showed an association between TT polymorphism and schizophrenia in Asian, European, and Chinese populations (Kheirollahi, Kazemi, & Ashouri, 2016), while another meta-analysis of 44 case–control studies found no association between the TT genotype and schizophrenia in Chinese, Caucasian and Japanese populations (Zhao et al., 2015).

*BDNF*-rs6265-T showed a highly anatomical replicated result among the primary studies – an association with reduction of hippocampal, parahipocampal and bilateral dorsolateral GM, of which only the superior frontal gyrus overlapped with the present study, though as a volume increase (at trend level; A-Bm-1/2Lat-Gen). In addition, a *BDNF*-rs6265-T-associated reduction of the left middle cingulate gyrus GM showed a high primary and present study concordance, albeit also at trend level in the latter. All this in a context of consistent GM reduction on the primary studies, where our study showed mostly increased volume in GM and decreased in WM.

The fact that a set of 10 primary studies served as basis for replication of the present one should have contributed to the extent of its primary and present study concordance. A few possible limitations might have contributed to decrease the degree of such concordance, namely the use of a proxy with r^2^ of (only) 0.8 in the present study, and the primary studies sample composition balanced towards healthy subjects and more diverse in terms of its ethnicity. Also, the present study showed no significant brain volume findings, and few at a trend level - yet none corresponding to the hippocampus, which was the most replicated among the primary studies.

#### ZNF804A-rs1344706

The *ZNF804A* gene is located in chromosome 2q32.1, consisting of four exons and encoding for the 1210 amino acid ‘zinc finger protein 804A’ of the zinc-finger protein family. SNP rs1344704 is located in the second intron of the *ZNF804A* gene, being the first to show a significant association with increased susceptibility to schizophrenia in a GWAS (O'Donovan et al., 2008), which has been consistently replicated in larger subsequent ones (Ripke et al., 2014; Steinberg et al., 2011; Williams et al., 2011), also extending to bipolar disorder, mainly in the context of European populations. ZNF804A is known to be highly expressed in brain tissues, specifically in the cytoplasm, neurites and dendritic spines in the human cortex (with higher density in the cingulate cortex), as well as in various types of mouse neurons (including pyramidal, dopaminergic and glutamatergic ones) (Dong, Mao, Chen, Yoon, & Mao, 2021).

In the present study, *ZNF804A*-rs11681373-A showed results only at trend level, with several findings overlapping with the primary studies anatomically, namely an association with volume reduction in angular gyrus GM and in rectus gyri WM (GM in primary studies). Nevertheless, our ROI analyses for this SNP were non-significant. Supporting the amount of replications found in the present study for this SNP is: (1) the fact that a proxy SNP highly correlated with the SNP featured in the primary studies has been used; (2) the sample composition regarding disease/condition among the primary studies is very similar to the present study one; (3) the SNP's risk derives from consistently replicated GWAS; and (4) a large set of six primary studies (corresponding to a large diversity of brain regions showing a genotype effect) was considered. On the other hand, 3 of the primary studies tested an effect of genotype on brain volume through slightly different measures than the present study (2 using cortical thickness, and 1 using WM density), which may have contributed to confound the replication results.

#### NRG1 And DISC1

The *NGR1* gene is located on chromosome 8p12 and encodes for a glycoprotein involved in neuronal survival, synaptogenesis, myelin formation, astrocytic differentiation and microglial activation. It plays a protective role in dopaminergic neurons (Prata et al., 2019a). Recent meta-analyses provided support for the association of *NRG1* with schizophrenia, although with a variety of haplotypes located throughout the gene (Li, Collier, & He, 2006; Munafò, Thiselton, Clark, & Flint, 2006; Tosato, Dazzan, & Collier, 2005). Rs6994992 is part of the original schizophrenia-associated risk HapICE haplotype (Stefansson et al., 2002) and has been found to be associated with altered transcription factor binding and increased levels of type IV *NRG1* mRNA in postmortem brain tissue (Law et al., 2006) and alteration in type IV promoter activity in *in vitro* receptor assays (W et al., 2007). The DISC1 protein interacts with proteins in the dopamine signalling pathway and is also associated with cortical development and neuronal migration (Kähler et al., 2012). Its gene was originally discovered on a breakpoint caused by a (1;11) (q42;q14.3) translocation found within a large Scottish family, whilst also being associated through linkage analysis with an increased prevalence of mental illness (also including psychotic disorders such as schizophrenia and bipolar disorder) (Blackwood et al., 2001). Its role in mental disorders is currently controversial (Porteous et al., 2014; Sullivan, 2013), with common variants within or near it showing non-significant associations with schizophrenia in a large GWAS (Ripke et al., 2014) and a meta-analysis of candidate and GWAS (Mathieson, Munafò, & Flint, 2012), while substantial evidence for its involvement in mental illness exists in family studies (Brzustowicz, Hodgkinson, Chow, Honer, & Bassett, 2000; Ekelund et al., 2004). Rs11122319 minor allele (‘risk allele’ in our study) is associated with cis-regulated gene expression (not tested in brain cells) (Carless et al., 2011). In addition, rs2793092 showed association with schizophrenia in a study with Taiwanese families (Liu et al., 2006).

In general, both SNPs in *NRG1* and *DISC1* presented very low primary and present study concordance. This could be explained by the relatively weak and ambiguous association of its risk alleles with schizophrenia, the relatively small set of studies reporting significant effects on brain volume (and the fact that they are more dispersed throughout different SNPs) and *NRG1*-rs35753505 having found significant genotype effects among patients only. Furthermore, analyses on the present study regarding *NRG1*-rs35753505 and *NRG1*-rs4733264 (proxy of rs6994992) were excluded due to likely confounding by age, *NRG1*-rs6994992 and *DISC1*-rs2793092 reported effects on ventricular volumes which the present study did not assess, and one primary study regarding *NRG1*-rs35753505 had its brain volume findings not adjusted for TIV (Cannon et al., 2012).

### General limitations

Aspects which may have contributed to lower primary and present study concordance include – in our study compared to primary studies, as well as in-between primary studies: (1) variation in whether SNPs were genotyped or imputated and SNP microarray coverage (e.g. genome-wide SNP arrays were used for the present review and only one of the selected SNPs was actually genotyped, with the remaining requiring proxies such as one with an *r*^2^ = 0.8 and the remaining five with a r^2^ average of 0.98); (2) some variation in diagnosis and volumetric measures used; (3) variation in MRI acquisition protocol and scanner; (4) whether adjustment for covariates (e.g. psychiatric medication) was made; (5) exclusion of analyses found to have true confounding variables (in the present study); and (5) unassessed epigenetic and gene-gene interactions involving both genotyped and non-genotyped SNPs with effects on brain volume. We note that the fact that our sample had the above heterogeneity limitations, although potentially increasing the statistical noise (which we tried to correct for), also increased its generalizability and representativeness of the studies we aimed to replicate, given that these also cut across diagnoses, volumetric measures, scanners and acquisition protocols. We also note that our concordance measure is limited by not being continuous, and thus not expressing subtle differences in concordance between findings. Nevertheless, future validation of our findings in a larger sample able to investigate each SNP effect in individual diagnostic groups, of imaging parameters, would be welcome. Lastly, although our sample's size may limit the detection of smaller effects, it is the same or larger than most studies we aimed to replicate.

## Conclusion

Seven psychosis-implicated SNPs across five different genes (proxies of *ZNF804A-*rs1344706, *CACNA1C-*rs1006737, *BDNF-*rs6265, *DISC1-*rs2793092, *DISC1-*rs11122319, *NRG1-*rs6994992 and *NRG1-*rs35753505 itself) were examined in our independent sample for effects on brain volume. A restricted number of replicated findings in the literature for such effects (measured through VBM) were further replicated in our sample. Concordance within previous literature was found, in decreasing order, for *BDNF-*rs6265, *ZNF804A-*rs1344706 and *CACNA1C-*rs1006737. Concerning our replication findings, concordance was found for the same genes, in the following decreasing order: a *CACNA1C-*rs1006737-A significant association with volume increase of DLPFC WM and right hippocampus, a *ZNF804A-*rs1344706-A ‘trend’ association with volume reduction in angular gyrus GM (AA < AG) and in rectus gyri WM (AA < G.car) and a *BDNF-*rs6265-T ‘trend’ association with volume change of superior frontal and cingulate gyrus GM. Although the degree of concordance among findings in prior literature – and in comparison with our study – was generally low, the detected replications could serve to instil more confidence in the involvement of *CACNA1C*, *BDNF* and ZNF804A genes in influencing brain structure. More studies are recommended to clarify inconsistencies in neuroimaging genetics results, especially using larger sample sizes, a careful account of ancestry heterogeneity, and meta or mega analyses of available data.

## Supplementary material

For supplementary material accompanying this paper visit https://doi.org/10.1017/S0033291722002896. click here to view supplementary material
