# Supplementary material for: Effects of psychosis-associated genetic markers on brain volumetry: a systematic review of replicated findings and an independent validation
Source: Psychol Med. 2022 Sep 28;52(16):3753–68. doi: 10.1017/S0033291722002896 (PMC9811278; doi:10.1017/S0033291722002896)
Supplement: Supplementary file 1 [file S0033291722002896sup001.doc]

**Supplementary material**

Index (Automatic)

[**Supplementary methods 3**](#__RefHeading___Toc106190040)

[**Sampling.** 3](#__RefHeading___Toc106190041)

[**Genotyping and imputation.** 4](#__RefHeading___Toc106190042)

[**Image acquisition.** 5](#__RefHeading___Toc106190043)

[**Image pre-processing.** 6](#__RefHeading___Toc106190044)

[**SNPs match to our sample.** 7](#__RefHeading___Toc106190045)

[**Neuroimaging genetics analysis.** 8](#__RefHeading___Toc106190046)

[**Effect of covariates on the SNP’s genotype and brain volume.** 10](#__RefHeading___Toc106190047)

[Supplementary results 12](#__RefHeading___Toc106190048)

[**Demographic and potential covariates analysis** 12](#__RefHeading___Toc106190049)

[**Table S1.** Main effect of SNP’s proxies genotypes on grey matter volume at whole-brain analysis 13](#__RefHeading___Toc106190050)

[**Table S2.** Main effect of SNP’s proxies genotypes on white matter volume at the whole-brain analysis. 16](#__RefHeading___Toc106190051)

[**Table S3.** Peak coordinates of where the effect of total intracranial volume on grey matter volume is maximum. 18](#__RefHeading___Toc106190052)

[**Table S4.** Peak coordinates where the effect of total intracranial volume on white matter volume is maximum. 18](#__RefHeading___Toc106190053)

[**Table S5.** Peak coordinates where the effect of age at scan on grey matter volume is maximum. 18](#__RefHeading___Toc106190054)

[**Table S6.** Peak coordinates where the effect of age at scan on white matter volume is maximum. 19](#__RefHeading___Toc106190055)

[**Table S7.** Peak coordinates where the effect of diagnosis on grey matter volume is maximum. 20](#__RefHeading___Toc106190056)

[**Table S8.** Peak coordinates where the effect of diagnosis on white matter volume is maximum. 21](#__RefHeading___Toc106190057)

[**Table S9.** Peak coordinates where the effect of handedness on grey matter volume is maximum. 21](#__RefHeading___Toc106190058)

[**Table S10.** Peak coordinates where the effect of handedness on white matter volume is maximum. 21](#__RefHeading___Toc106190059)

[**Table S11.** Peak coordinates where the effect of MRI scan acquisition protocol on grey matter volume is maximum. 22](#__RefHeading___Toc106190060)

[**Table S12.** Peak coordinates where the effect of MRI scan acquisition protocol on white matter volume is maximum. 23](#__RefHeading___Toc106190061)

[**Table S13.** Peak coordinates where the effect of sex on grey matter volume is maximum. 24](#__RefHeading___Toc106190062)

[**Table S14.** Peak coordinates where the effect of sex on white matter volume is maximum. 24](#__RefHeading___Toc106190063)

[**Table S15.** Association analyses between age at scan, sex and handedness and each SNP’s proxy genotype. 24](#__RefHeading___Toc106190064)

[**Table S16.** Association analysis between subject’s diagnosis and genotype. 26](#__RefHeading___Toc106190065)

[**Table S17.** Association analysis between MRI scanning protocol and genotype. 28](#__RefHeading___Toc106190066)

[**Table S18.** Association analysis between total intracranial volume and genotype. 30](#__RefHeading___Toc106190067)

[**Table S19.** Excluded studies assessing a SNP genotype effect on brain volume, with reasons for exclusion. 32](#__RefHeading___Toc106190068)

[**Table S20.** Demographics of the present study’s sample. 34](#__RefHeading___Toc106190069)

[**Table S21.** Sample size, diagnostic and ancestry composition, per included primary study. 35](#__RefHeading___Toc106190070)

[**Table S22.** DISC1 and NRG1: primary studies of its SNPs of interest and their corresponding effects on brain volume 36](#__RefHeading___Toc106190071)

[**Figure S1.** Effect of total intracranial volume on gray (left) and white (right) matter volumes. 37](#__RefHeading___Toc106190072)

[**Figure S2.** Effect of age at scan on grey (left) and white (right) matter volumes. 37](#__RefHeading___Toc106190073)

[**Figure S3.** Effect of diagnosis on grey (left) and white (right) matter volumes. 37](#__RefHeading___Toc106190074)

[**Figure S4.** Effect of handedness on grey (left) and white (right) matter volumes. 38](#__RefHeading___Toc106190075)

[**Figure S5.** Effect of MRI scan protocol on grey (left) and white (right) matter volumes. 38](#__RefHeading___Toc106190076)

[**Figure S6.** Effect of sex on grey (left) and white (right) matter volumes. 38](#__RefHeading___Toc106190077)

[**Figure S7.** Population stratification analysis. 39](#__RefHeading___Toc106190078)

[References 40](#__RefHeading___Toc106190079)

# Supplementary methods

**Sampling.** We examined a total sample of 113 subjects, including 14 patients with schizophrenia and 21 with bipolar disorder, 34 with an ARMS (9 who later transitioned to psychosis, ARMS-T, and 25 who did not, ARMS-NT), and 44 healthy volunteers. Schizophrenia and bipolar patients, and healthy volunteers were recruited from the South London and Maudsley (SLaM) National Health Service (NHS) Foundation Trust as part of the National Institute of Health Research (NIHR) Biomedical Research Centre (BRC) Genetics and Psychosis (GAP) study conducted in South London, UK^1^. Diagnosis, according to the Diagnostic and Statistical Manual of Mental Disorders (DSM) fourth Edition^2^, was ascertained by an experienced psychiatrist using a structured diagnostic interview with instruments detailed elsewhere^3^. All schizophrenia and bipolar patients were in a stable clinical state when participating. The study was approved by the National Health Service South East London Research Ethics Committee, UK (Project “Genetics and Psychosis (GAP)” reference number 047/04). All subjects gave written informed consent. ARMS individuals were recruited from the Outreach And Support In South London (OASIS) high-risk service, SLaM NHS Foundation Trust^4^, and assessed using the comprehensive assessment of the ARMS (CAARMS)^5^, whereby they meet criteria if they: 1) had a recent decline in psychosocial function coupled with either schizotypal personality disorder or a first degree relative with psychosis, examined using the family interview for genetic studies (FIGS)^6^; or 2) experienced attenuated positive psychotic symptoms; or 3) had a Brief Limited Intermittent Psychosis (BLIP) episode lasting less than one week which resolved without antipsychotics. Exclusion criteria applied to all participants were: 1) history of significant head injury and current (last 12 months) substance dependency according to DSM-IV diagnostic criteria, and 2) non-European ancestry.

***Genotyping and imputation.*** DNA was extracted from blood or cheek swabs. The samples were genotyped either at the SLaM NHS Foundation Trust/King’s College London BRC Genomics Laboratory on the Illumina HumanCore Exome BeadChip (“SLaM sample”, 59 subjects – 9 ARMS-T, 25 ARMS-NT, 9 schizophrenia patients and 16 healthy subjects) or at the Wellcome Trust Sanger Institute (WTSI, Cambridge, UK) on the Genome-wide Human Single Nucleotide Polymorphism (SNP) Array 6.0 (“WTSI sample”, 54 subjects – 5 schizophrenia, 21 bipolar patients, 28 healthy subjects). Quality control (QC) included exclusion of SNPs with minor allele frequency (MAF) <1% or 2%, SNPs with genotypic failure >1% or >5%, and individuals with genotypic failure >1% or 2%, and SNPs with Hardy-Weinberg equilibrium p<10-5 in healthy subjects only or p<10-6 in SLaM or WTSI sample, respectively. Imputation was performed with IMPUTE2^7^ based on the 1000 Genomes phase 3 reference panel (19). The imputed markers underwent a second stage of QC to exclude SNPs that were missing in >5% or 1% of individuals in SLaM or WTSI sample, respectively, or had imputation information score (INFO) <0.8.

SLaM and WTSI samples were merged keeping only overlaped imputed SNPs. To account for genotyping and imputation QC differences in the two samples and following standard Genome-wide association studies (GWAs) QC guidelines^8^, an extra QC was run excluding SNPs missing in >2% of individuals and with a MAF <5%. Samples merging and QC were conducted using PLINK 1.9^9^.

***Image acquisition.*** Structural MRI scans were acquired with two different scanners using eight different protocols: a) acquisition protocol 1 – enhanced fast gradient echo 3-Dimensional (efgre3D) sequence, 1.5T Signa scanner (General Electric Medical Systems, USA; voxel size = 0.9 x 0.9 x 1.5 mm^3^; matrix of acquisition = 256 x 256 x 124; field-of-view = 240 mm; gap = 0 mm; repetition/echo/inversion times = 8.7 ~ 9.1 s/1.8 ~ 2 s/450 s; flip angle = 20°; 14 scans: 5 schizophrenia patients (SCZ) and 9 controls; b) acquisition protocol 2 – efgre3D sequence, 1.5T Signa scanner (General Electric Medical Systems, USA; voxel size = 0.9 x 0.9 x 1.5 mm^3^; matrix of acquisition = 256 x 256 x 124; field-of-view = 240 mm; gap = 0 mm; repetition/echo/inversion times = 15 s/1.8 s/450 s; flip angle = 20°; 9 scans: 4 SCZ and 5 controls; c) acquisition protocol 3 – efgre3D sequence, 1.5T Signa scanner (General Electric Medical Systems, USA; voxel size = 0.9 x 0.9 x 1.5 mm^3^; matrix of acquisition = 256 x 256 x 124; field-of-view = 240 mm; gap = 0 mm; repetition/echo/inversion times = 17.8 ~ 18 s/5 ~ 5.1 s/450 s; flip angle = 20°; 38 scans: 3 SCZ, 14 bipolar patients (BP), 13 relatives, and 8 controls; d) acquisition protocol 4 – efgre3D sequence, 1.5T Signa scanner (General Electric Medical Systems, USA; voxel size = 0.9 x 1.5 x 0.9 mm^3^; matrix of acquisition = 256 x 124 x 256; field-of-view = 220 mm; gap = 0 mm; repetition/echo/inversion times = 11.9 ~ 13.1 s/5.2 ~ 5.8 s/450 s; flip angle = 20°; 68 scans: 6 SCZ, 14 BP, 16 relatives, and 32 controls; e) acquisition protocol 5 – Gradient Recalled Acquisition in Steady State (grass) sequence, 1.5T Signa scanner (General Electric Medical Systems, USA; voxel size = 0.8 x 1.5 x 0.8 mm^3^; matrix of acquisition = 256 x 124 x 256; field-of-view = 200/240 mm; gap = 0 mm; repetition/echo/inversion times = 35 s/5 s/0 s; flip angle = 35°; 6 scans: 1 SCZ, and 5 controls; f) acquisition protocol 6 – efgre3d sequence, 1.5T Signa scanner (General Electric Medical Systems, USA; voxel size = 0.9 x 0.9 x 1.5 mm^3^; matrix of acquisition = 256 x 256 x 124; field-of-view = 220 mm; gap = 0 mm; repetition/echo/inversion times = 15.9 s/5.2 s/300 s; flip angle = 20°; 19 scans: 3 patients at an at-risk mental state that later transitioned to psychosis (ARMS-T), and 16 ARMS-NT; g) acquisition protocol 7 – efgre3d sequence, 1.5T Signa scanner (General Electric Medical Systems, USA; voxel size = 0.9 x 0.9 x 1.5 mm^3^; matrix of acquisition = 256 x 256 x 124; field-of-view = 220 mm; gap = 0 mm; repetition/echo/inversion times = 21.3 s/5.1 s/0 s; flip angle = 20°; 33 scans: 14 ARMS-T, and 19 ARMS-NT; and h) acquisition protocol 8 – efgre3d sequence, 3T Signa scanner (General Electric Medical Systems, USA; voxel size = 1.1 x 1.1 x 1.1 mm^3^; matrix of acquisition = 256 x 256 x 146~196; field-of-view = 280 mm; gap = 1.1 mm; repetition/echo/inversion times = 6.6~7.2 s/2.8~2.9 s/450 s; flip angle = 20°; 47 scans: 6 ARMS-T, and 41 ARMS-NT.

***Image pre-processing.*** Structural magnetic ressonance imaging (MRI) scans were acquired with two different scanners using eight different protocols. T1-weighted images were free of artifacts (assessed through visual inspection) and processed with CAT12 (v1092 (Jena, Germany)^10^, a SPM12 add-on (v6909) (London, UK)^11^ using default settings and MATLAB (9.1) (Natick, Massachussetts)^12^. Firstly, bias field inhomogeneity correction was performed. Secondly, images were segmented into GM, WM, and cerebrospinal fluid. Thirdly, images were spatially normalized to a template from the IXI dataset (London, UK)^13^ using the DARTEL^14^ algorithm. Finally, Jacobian scaled (“modulated”) warped tissue maps were then created for both GM and WM, and the resultant images were then smoothed with an 8×8×8 mm Gaussian kernel. Moreover, the total intracranial volume was computed by summing all voxels classified as GM or WM or as cerebrospinal fluid.

**SNPs retrieved through systematic review and analysed in our independent sample**

***SNPs match to our sample.*** SNP selection in our sample was done using the PLINK sofware (version 1.9). The ARMS and GAP samples were first modeled to fit the typical population from which the SNPs of interest were obtained (mostly “White Europeans/North-americans”). Although this ethnical label was assigned through self-classification in our sample, we validated it through a population stratification analysis using principal component analysis. The search for the SNPs was done using their location in the genome. As only one match was obtained (SNP *rs35753505*, of the *NRG1* gene), we sought to select appropriate proxys for the remaining unmatched SNPs, to be found in the subjects database. Criteria for an appropriate proxy were defined as: a) having an r^2^>0.8 (measure of linkage disequilibrium) with the SNP of interest and b) being present in the subjects database. The proxy selection was performed through the linkage disequilibrium-link (LD-link) web-based application^15^. Again using PLINK, proxys with the highest r^2^ for each SNP of interest were searched in the subjects database. 17 proxys (variably corresponding to the nine previously unmatched SNPs of interest) were found. Following the application of the r^2^>0.8 criterion, and by randomly selecting only one proxy per SNP when more than one of the resulting proxys had the same r^2^, 11 proxies were excluded, which also meant the exclusion of 3 SNPs of interest. The set of SNPs of interest retrieved from the primary studies combined with this SNP matching process to our sample meant that, for each gene, only one SNP of interest was selected (except in the case of *DISC1* and *NRG1*, where two SNPs were selected, given that both were either found or had adequate proxys found within our sample). Next, the haplotypes of these matched and selected SNPs were determined, in our sample. Through LD-link, the risk alleles of these proxy SNPs were matched with the risk alleles of the SNPs primarily selected, from the individual studies. Genotype was missing for *rs11681373*2, for 1 healthy subject.

***Neuroimaging genetics analysis.*** The effect each SNP’s genotype had on GM and WM volume was tested using a whole-brain analysis, and a region-of-interest (ROI) analysis. For the whole-brain analysis, we have run an analysis of variance (ANOVA) with the voxel-based GM and WM volume maps as dependent variables (i.e. one statistical model per brain tissue type) and the subjects’ SNP genotype as independent variable (i.e. one statistical model per SNP). Genotypes were coded according to one of the following genetic models: a) dominant for “risk” allele (i.e. whereby “risk” allele homozygous and heterozygous were contrasted against “non-risk” allele homozygous); b) dominant for “non-risk” allele (i.e. whereby “non-risk” allele homozygous and heterozygous were contrasted against “risk” allele homozygous); or c) additive (i.e. whereby homozygous for “risk” allele were contrasted against heterozygous and against homozygous for “non-risk” allele, and heterozygous were also contrasted against homozygous “non-risk” allele ). For each SNP, classification of alleles as “risk/nonrisk” and the choice of genotype effect model was made entirely based on the allele classification and model used by the primary studies from which the SNPs were selected. When different models had been used for each SNP, all were tested. For completeness, for each SNP model, we tested for a “negative” (decreasing volume) and a “positive” (increasing volume) effect of genotype on brain volume in whole-brain analyses. Furthermore, the following variables were included in the main statistical model as covariates of no interest: age at the time of brain scan acquisition, sex (i.e. female, male), handedness (i.e. right-handed, left-handed or ambidextrous), diagnosis (i.e. schizophrenia, bipolar disorder, ARMS-T, ARMS-NT, and healthy controls), total intracranial volume (TIV), and scan protocol (eight different acquisition protocols).

To achieve a more precise validation (closer to a *de facto* replication) of previously reported SNP’s genotype effects on brain volume, we conducted ROI analyses using the same design for each SNP as for the whole-brain analysis. For this, we collected the reported peaks of genotype effect from the primary studies and built individual masks from them (one per peak) using the Wake Forest University (WFU) pickatlas SPM12 add-on^16^. Moreover, masks were defined as: a) spheres with a radius of 10 mm^17^, if the peak coordinates were available in the primary studies; or b) brain regions defined by the automated anatomical labeling atlas (AAL)^18^, for testing reported effects on GM or WM volume, unless otherwise stated, if the peak coordinates were not available. In instances where the primary study reported effects on cortical thickness, we applied an AAL-defined mask on GM volume. Whenever no AAL region available corresponded exactly to the one reported in the primary study, we sought to use the most anatomically approximate one. No ROI analysis was conducted for a SNP model for which the primary study reported significant effects on ventricular volumes. In all ROI analyses, we tested only for effects in the direction reported by the primary studies, given the high hypothesis-driven context.

All whole-brain and ROI statistical analyses were defined using a full factorial design using SPM12 and its add-on CAT12. Moreover, the effect of each SNP’s genotype on GM or WM volume was considered to be statistically significant: a) at a whole-brain level, at p-value < .05 after correction for multiple testing (i.e. for the number of voxels), with no cluster size cut-off, using a voxel-level Family Wise Error Rate (FWER) and b) on a ROI level, at p-value <.001 uncorrected, also with no cluster size cut-off. All other effects with an uncorrected p-value < .001, and exceding a cluster size cut-off of 25 voxels, are reported as ‘trends’. In all analyses, significant and trend results were mapped anatomically using the “xjview” toolbox (<https://www.alivelearn.net/xjview>) and, in the case of large resulting clusters (>200 voxels), further confirmed manually through printed book atlases. All mentions of genotype comparisons, either from the primary studies or the present study, both in the manuscript, tables and figures, are stated as “high-risk allele load vs. low-risk allele load”.

The assessment of how much of the interindividual variance in regional brain volume was explained by the genetic variation was determined through the partial eta squared measure: for each analysis where significant SPM results were found (either at FWER-corrected in whole-brain or uncorrected in ROI), the volumes of grey or white matter per subject where extracted at the voxel of peak effect (according to the contrast comparing the genotypes of interest). Then those volumes were entered as a dependent variable in a univariate GLM model in SPSS, to estimate the effect of “genotype”, entering in the model the same covariates of no interest as previously in the SPM models (either as fixed factors or continuous covariates).

***Effect of covariates on the SNP’s genotype and brain volume.*** In order to identify potential demographic and imaging processing extraneous and confounding variables in our imaging genetics analyses, we performed a two-step association analysis. First, we tested the statistical association between each of these variables and the genotype for each SNP included in the study (i.e. the independent variable in our main statistical model) (Tables S15-S18 for details). For categorical variables (i.e. sex, handedness, MRI scan acquisiton protocol, diagnostic group) a chi-square or an Fischer’s exact (if at least one of the crosstable cells had less than 5 observations) test were used. For continous variables (i.e. age and total intracranial volume) two-sample t-test (for dominant type models) or one-way ANOVA (for additive type models) was used. Second, we tested the effect of each of these potential extraneous and confounding variables on brain grey and white matter volumes (i.e. the dependent variable in our main statistical model) (Tables S3-S14 and Figures S1-S6). Each potential extraneous and confounding variable is assigned as a) a true extraneous variable if it is associated with (i.e. at an uncorrected p-value < .05) brain volume changes (either in grey or white matter), but not with the genotype of a given SNP ; b) a true confounder variable if it is associated with the genotype of a given SNP and has also a significant effect on brain volume; and c) a non extraneous variable if it is neither associated with the genotype of a given SNP, nor has an effect on brain volume. Only variables classified as true extraneous variables were included in the main statistical model as covariates of no interest. Analyses shown to have “true confounder variables” were excluded (see next topic for details). All non-imaging statistical analyses were conducted in SPSS (IBM Corp. Released 2017. IBM SPSS Statistics for Windows, Version 25.0. Armonk, NY: IBM Corp); the imaging-related statistical analyses were conducted in SPM12^11^ and its add-on CAT12. ^10^

# Supplementary results

***Demographic and potential covariates analysis***

Since for analyses all related to *NRG1* *rs35753505* and *rs4733264* age was found to be, by chance, significantly associated with these SNPs genotypes and for analyses relating to A carriers vs. GG in *ZNF804A* *rs11681373*1 scan protocol was found, also by chance, to be associated with this SNP genotype in our sample (both being, therefore, potential confounders in the respective models) those analyses were fully excluded. No other associations between covariates and “genotype” independent variable were detected. For complete information on these analyses, see tables S16-S19.

**Table S1. Main effect of SNP’s proxies genotypes on grey matter volume at whole-brain analysis. Results at ‘trend’ level (i.e. uncorrected p-value < .001) and in bold statistically significant at FWE-corrected level. A voxel cluster size of “25” was applied to “trend” results.**

| Gene**/**SNP/**Proxy**  (Proxy genotype coding)  (Risk genotype on the left) | **Cluster size^1^**  (voxels) | **F** | **Z** | **unc-p** | **FWER-p** | **x**  (mm) | **y**  (mm) | **z**  (mm) | **Main regions**  (aal)^2^ | **Effect’s direction** |
| --- | --- | --- | --- | --- | --- | --- | --- | --- | --- | --- |
| DISC1 rs11122319  **rs1417585**  (CC *vs.* CT *vs.* TT) | 58 | 10.13 | 3.71 | < .001 | .721 | 4.5 | -51 | -63 | undefined | CT < TT |
|  | 46 | 9.57 | 3.59 | < .001 | .845 | -7.5 | -49.5 | -63 | undefined | CT < TT |
|  | 54 | 9.41 | 3.56 | < .001 | .875 | 1.5 | -69 | 37.5 | Precuneus_L | CC > TT |
| DISC1 rs2793092  **rs2793098**  (AA *vs.* G-car.**)** | n.s. |  |  |  |  |  |  |  |  |  |
| DISC1 rs2793092  **rs2793098**  (AA *vs.* AG *vs.* GG) | 252 | 20.21 | 4.11 | < .001 | .291 | 46.5 | -18 | -12 | Temporal Mid_R | AA>GG; AG>GG |
|  | 187 | 17.77 | 3.86 | < .001 | .565 | -7.5 | -15 | 18 | Ventral Anterior Nucleus // Thalamus_L | AA<GG; AG<GG |
|  | 199 | 17.34 | 3.81 | < .001 | .623 | 13.5 | -19.5 | 16.5 | Lateral Posterior Nucleus // Thalamus_R | AG<GG; AA<GG |
|  | 72 | 17.02 | 3.78 | < .001 | .665 | 24 | 52.5 | 39 | Frontal_Sup_R | AG<GG; AA>AG |
|  | 180 | 16.05 | 3.67 | < .001 | .791 | 58.5 | -9 | 16.5 | R_Oper_Portion_Inf_Frontal | AG<GG; AA<GG |
| ZNF804A rs1344706  **rs11681373**  (AA vs. G-car.) | n.s. |  |  |  |  |  |  |  |  |  |
| ZNF804A rs1344706  **rs11681373**  (AA *vs.* AG *vs.* GG) | 126 | 10.28 | 3.74 | < .001 | .767 | 39 | -63 | 54 | Angular_R | AG>GG |
|  | 64 | 10.02 | 3.68 | < .001 | .823 | -1.5 | -87 | -7.5 | Calcarine_L | AG<GG |
|  | 39 | 9.69 | 3.62 | < .001 | .884 | -27 | -85.5 | -6 | Occipital_Inf_L | AG<GG |
|  | 35 | 8.71 | 3.40 | < .001 | .984 | 54 | 7.5 | 43.5 | Precentral_R | AG<GG |
| NRG1 rs35753505  (C-car. *vs.* TT) | 379 | 20.02 | 4.10 | < .001 | .286 | 46.5 | -1.5 | 51 | Frontal_Mid_R | C-car. < TT |
|  | 467 | 18.63 | 3.95 | < .001 | .429 | 52.5 | 4.5 | -18 | Temporal_Pole_Mid_R | C-car. < TT |
|  | 118 | 18.47 | 3.94 | < .001 | .448 | -6 | -37.5 | 45 | Cingulum_Mid_L | C-car. < TT |
|  | 220 | 18.18 | 3.91 | < .001 | .484 | 16.5 | 58.5 | 12 | Frontal_Sup_Medial_R | C-car. < TT |
|  | 97 | 18.07 | 3.89 | < .001 | .497 | -1.5 | 60 | 1.5 | Frontal_Sup_Medial_L | C-car. < TT |
|  | 249 | 17.20 | 3.80 | < .001 | .612 | 43.5 | -18 | -9 | Temporal Lobe_Sub-gyral_R | C-car. < TT |
|  | 543 | 17.16 | 3.79 | < .001 | .617 | -37.5 | 33 | 6 | Frontal_Inf_Tri_L | C-car. < TT |
|  | 25 | 15.35 | 3.59 | < .001 | .846 | 31.5 | 64.5 | 12 | Frontal_Sup_R | C-car. < TT |
|  | 46 | 15.35 | 3.59 | < .001 | .847 | 30 | -1.5 | 61.5 | Frontal_Sup_R | C-car. < TT |
|  | 130 | 15.20 | 3.57 | < .001 | .861 | 61.5 | -21 | -3 | Temporal_Sup_R | C-car. < TT |
|  | 282 | 14.84 | 3.53 | < .001 | .896 | -37.5 | 21 | -9 | Frontal_Inf_Orb_L | C-car. < TT |
|  | 41 | 14.64 | 3.50 | < .001 | .912 | 42 | 34.5 | 25.5 | Frontal_Inf_Tri_R | C-car. < TT |
|  | 51 | 13.78 | 3.39 | < .001 | .965 | -58.5 | -45 | 1.5 | Temporal_Mid_L | C-car. < TT |
|  | 30 | 13.26 | 3.33 | < .001 | .982 | -6 | -73.5 | -33 | Cerebelum_Crus2_L | C-car. < TT |
| NRG1 rs6994992  **rs4733264**  (G-car. *vs.* CC) | 594 | 19.13 | 4.00 | < .001 | .379 | -42 | 30 | 12 | Frontal_Inf_Tri_L | G-car. < CC |
|  | 185 | 16.95 | 3.77 | < .001 | .653 | 48 | 0 | 52.5 | Frontal_Mid_R | G-car. < CC |
|  | 412 | 16.91 | 3.77 | < .001 | .658 | -58.5 | -34.5 | -9 | Temporal_Mid_L | G-car. < CC |
|  | 63 | 15.72 | 3.63 | < .001 | .810 | 51 | -60 | 25.5 | Angular_R | G-car. < CC |
|  | 342 | 15.68 | 3.63 | < .001 | .815 | 34.5 | -36 | -24 | Fusiform_R | G-car. < CC |
|  | 86 | 15.56 | 3.61 | < .001 | .828 | 43.5 | 3 | -28.5 | Temporal Mid_R | G-car. < CC |
|  | 77 | 15.35 | 3.59 | < .001 | .851 | -3 | 61.5 | 1.5 | Frontal_Sup_Medial_L | G-car. < CC |
|  | 90 | 15.07 | 3.55 | < .001 | .879 | 18 | 40.5 | 36 | Frontal_Sup_R | G-car. < CC |
|  | 120 | 14.99 | 3.54 | < .001 | .887 | 18 | 60 | 18 | Frontal_Sup_R | G-car. < CC |
|  | 50 | 14.67 | 3.50 | < .001 | .914 | -13.5 | -39 | -3 | Lingual_L | G-car. < CC |
|  | 69 | 14.62 | 3.50 | < .001 | .917 | 42 | -18 | -9 | Temporal Lobe_Sub-Gyral_R | G-car. < CC |
|  | 239 | 14.57 | 3.49 | < .001 | .921 | -36 | 19.5 | -12 | Insula_L | G-car. < CC |
|  | 39 | 14.55 | 3.49 | < .001 | .923 | 60 | -43.5 | 6 | Temporal_Mid_R | G-car. < CC |
|  | 41 | 13.74 | 3.39 | < .001 | .968 | -28.5 | 21 | 57 | Frontal_Mid_L | G-car. < CC |
|  | 38 | 13.06 | 3.30 | < .001 | .988 | -4.5 | 48 | -19.5 | Orbital Gyrus_Rectus_L | G-car. < CC |
|  | 27 | 12.26 | 3.19 | 0,001 | .997 | 24 | -75 | -46.5 | Cerebellum_Inf Semi-Lunar Lobule_R | G-car. < CC |
| NRG1 rs6994992  **rs4733264**  (GG *vs.* GC *vs.* CC) | 127 | 16.79 | 3.75 | < .001 | .667 | -18 | 15 | 64.5 | Frontal_Sup_L | GG>GC |
|  | 52 | 15.20 | 3.57 | < .001 | .860 | 66 | -46.5 | 25.5 | SupraMarginal_R | GG<GC |
| BDNF rs6265  **rs4923457**  (T-car. *vs.* AA) | 35 | 13.70 | 3.38 | < .001 | .976 | -51 | -22.5 | 31.5 | Postcentral_L | T-car. > AA |
| BDNF rs6265  **rs4923457**  (TA *vs.* AA) | 37 | 18.58 | 3.94 | < .001 | .477 | -30 | -91.5 | 27 | Cuneus_L | TA<AA |
|  | 25 | 15.06 | 3.54 | < .001 | .902 | 18 | 52.5 | 19.5 | Frontal_Sup_R | TA>AA |
|  | 40 | 14.20 | 3.44 | < .001 | .959 | -6 | -40.5 | 48 | Cingulum_Mid_L | TA>AA |
| CACNA1C rs1006737  **rs769087**  (AA *vs.* AG *vs.* GG) | 52 | 15.22 | 3.57 | < .001 | .882 | 15 | -76.5 | 13.5 | Calcarine_R | AA>AG |
|  | 32 | 13.11 | 3.30 | < .001 | .990 | -24 | -70.5 | -7.5 | Fusiform_L | AA<AG |

* significant after FWER-correction, but only for the peak of effect (peak with a cluster size of 2 voxels on the genotype main effect F-test)

^1^ A voxel cluster size cut-off of “25” was applied to “trend” results.

^2^ These labels were extracted with the “xjview” toolbox, that uses the AAL atlas to ascertain and describe the brain regions where the significant clusters stand; manual revision using printed atlases was also performed, for resulting clusters > 200 voxels.

(Abbreviations, in alphabetical order): car. = Carriers; Inf = Inferior; L = Left; Lat = Lateral; Mid = Middle; n.s. = non-significant (uncorrected p-value >.001); Oper = Opercular; Orb = Orbital; Post = Posterior; R = Right; Sup = Superior; Tri = Triangular.

**Table S2.** Main effect of SNP’s proxies genotypes on white matter volume at the whole-brain analysis. Results at ‘trend’ level (i.e. uncorrected p-value < .001) and in bold statistically significant at FWE-corrected level.

| Gene/SNP/**Proxy**  (Proxy genotype coding)  (Risk genotype on the left) | **Cluster size**  (voxels)^1^ | **F** | **Z** | **unc-p** | **FWER-p** | **x** (mm) | **y** (mm) | **z** (mm) | **Main regions**  (aal)^2^ | **Effect’s direction** |
| --- | --- | --- | --- | --- | --- | --- | --- | --- | --- | --- |
| DISC1 rs11122319  **rs1417585**  (CC vs. CT *vs.* TT) | n.s |  |  |  |  |  |  |  |  |  |
| DISC1 rs2793092  **rs2793098**  (AA *vs.* G-car.) | 135 | 22.39 | 4.32 | < .001 | .099 | 46.5 | -39 | 42 | Supramarginal_R | AA > G-car. |
| DISC1 rs2793092  **rs2793098**  (AA *vs.* AG *vs.* GG) | 157 | 19.16 | 4.00 | < .001 | .290 | 49.5 | -39 | 0 | Temporal_Mid_R | AG>GG; AA>GG |
|  | 63 | 17.85 | 3.87 | < .001 | .425 | 22.5 | 45 | 33 | Frontal_Sup_R | AG<GG |
| ZNF804A rs1344706  **rs11681373**  (AA *vs.* G-car.) | 70 | 19.12 | 4.00 | < .001 | .294 | 43.5 | -73.5 | -12 | Occipital_Inf_R | AA < G-car. |
|  | 77 | 14.39 | 3.47 | < .001 | .864 | -22.5 | -36 | 58.5 | Postcentral_L | AA < G-car. |
|  | 27 | 13.71 | 3.38 | < .001 | .923 | -10.5 | -46.5 | 63 | Precuneus_L | AA < G-car. |
|  | 29 | 13.40 | 3.34 | < .001 | .944 | 36 | -4.5 | -10.5 | Sub-lobar_ Extra-Nuclear_R | AA < G-car. |
|  | 30 | 12.60 | 3.24 | .001 | .979 | -12 | 31.5 | -18 | Rectus_L | AA < G-car. |
| ZNF804A rs1344706  **rs11681373**  (AA *vs.* AG *vs.* GG) | 91 | 13.27 | 4.30 | < .001 | .123 | 43.5 | -73.5 | -12 | Occipital_Inf_R | AA<GG |
|  | 73 | 9.22 | 3.51 | < .001 | .869 | -13.5 | 37.5 | -21 | Frontal_Sup_Orb_L | AA<AG |
| NRG1 rs35753505  (C-car. *vs.* TT) | 38 | 13.15 | 3.31 | < .001 | .953 | 18 | -3 | 51 | Frontal Med_R | C-car. < TT |
| NRG1 rs6994992  **rs4733264**  (G-car. *vs.* CC) | 80 | 18.7 | 4.0 | < .001 | .329 | -31.5 | 46.5 | 13.5 | Frontal_Mid_L | G-car. < CC |
|  | 105 | 14.8 | 3.5 | < .001 | .806 | 34.5 | -63 | 18 | Temporal Mid_R | G-car. < CC |
| NRG1 rs6994992  **rs4733264**  (GG *vs.* GC *vs.* CC) | n.s. |  |  |  |  |  |  |  |  |  |
| BDNF rs6265  **rs4923457**  (T-car. *vs.* AA) | 46 | 20.22 | 4.11 | < .001 | .204 | -45 | -33 | 42 | Parietal_Inf_L | T-car. > AA |
| BDNF rs6265  **rs4923457**  (TA *vs.* AA) | 263 | 16.32 | 3.69 | < .001 | .635 | 31.5 | -52.5 | 9 | Sup_Longitudinal_Fasciculus_R | TA<AA |
| CACNA1C rs1006737  **rs769087**  (A-car. *vs.* GG) | 25 | 16.21 | 3.69 | < .001 | .642 | 43.5 | -28.5 | -21 | Temporal_Inf_R | A-car. < GG |
|  | 60 | 14.85 | 3.52 | < .001 | .817 | -6 | 57 | -4.5 | Frontal_Med_Orb_L | A-car. < GG |
| CACNA1C rs1006737  **rs769087**  (AA *vs.* AG *vs.* GG) | **1455** | **26.24** | **4.66** | **< .001** | **.026*** | **-31.5** | **-21** | **-13.5** | **Hippocampus_L** | **AA>AG;AA>GG** |
|  | **2249** | **25.36** | **4.58** | **< .001** | **.036*** | **-4.5** | **-7.5** | **-1.5** | **Thalamus_L + R, Hippocampus R** | **AA>AG;AA>GG** |
|  | 265 | 20.03 | 4.09 | < .001 | .220 | 21 | -66 | 19.5 | Cuneus_R | AA>AG |
|  | 115 | 17.55 | 3.83 | < .001 | .460 | -48 | -37.5 | -1.5 | Middle Temporal Gyrus_L | AA>AG |
|  | 53 | 17.02 | 3.78 | < .001 | .526 | 25.5 | -63 | -6 | Fusiform_R | AA>AG |
|  | 144 | 16.76 | 3.75 | < .001 | .560 | -42 | -24 | 31.5 | Postcentral Gyrus_L | AA>AG |
|  | 31 | 16.34 | 3.70 | < .001 | .618 | 31.5 | -90 | -12 | Occipital_Inf_R | AA>AG |
|  | 171 | 15.16 | 3.56 | < .001 | .774 | -21 | -13.5 | 51 | Frontal Mid_L | AA>AG |
|  | 222 | 15.06 | 3.55 | < .001 | .786 | 16.5 | 22.5 | 19.5 | Caudate_nucleus_head R | AA>AG;AG<GG |
|  | 105 | 15.00 | 3.54 | < .001 | .794 | -9 | 33 | 3 | Frontal Lobe_Sub-Gyral_L | AA>AG;AG<GG |
|  | 74 | 14.68 | 3.50 | < .001 | .831 | -18 | -64.5 | 37.5 | Occipital_Sup_L | AA<AG;AA<GG |
|  | 44 | 13.28 | 3.33 | < .001 | .949 | -13.5 | 1.5 | 36 | Cingulate Gyrus_L | AA>AG |

* significant after FWER-correction, but only for the peak of effect (both peaks with a cluster size of 6 voxels on the genotype main effect F-test)

^1^ A voxel cluster size cut-off of “25” was applied to “trend” results.

^2^ These labels were extracted with the “xjview” toolbox, that uses the AAL atlas to ascertain and describe the brain regions where the significant clusters stand; manual revision using printed atlases was also performed, for resulting clusters > 200 voxels.

(Abbreviations, in alphabetical order): car. = Carriers; Inf = Inferior; L = Left; Lat = Lateral; Med = Medial; Mid = Middle; n.s. = non-significant (uncorrected p-value >.001); Oper = Opercular; Orb = Orbital; Post = Posterior; R = Right; Sup = Superior; Tri = Triangular

| Cluster number (size in voxels) | FWE corrected p | FDR corrected p | F | equivZ | uncorrected p | x (mm) | y (mm) | z (mm) |
| --- | --- | --- | --- | --- | --- | --- | --- | --- |
| 1 (368903) | < .001 | < .001 | 135.0 | 65535 | < .001 | 25.5 | -7.5 | -28.5 |

**Table S3.** Peak coordinates of where the effect of total intracranial volume on grey matter volume is maximum.

**Table S4.** Peak coordinates where the effect of total intracranial volume on white matter volume is maximum.

| Cluster size (voxels) | FWE corrected p | FDR corrected p | F | equivZ | uncorrected p | x (mm) | y (mm) | z (mm) |
| --- | --- | --- | --- | --- | --- | --- | --- | --- |
| 268707 | < .001 | < .001 | 265.5 | 65535 | < .001 | -15 | 22.5 | 7.5 |

**Table S5.** Peak coordinates where the effect of age at scan on grey matter volume is maximum.

| Cluster size (voxels) | FWE corrected p | FDR corrected p | F | equivZ | uncorrected p | x (mm) | y (mm) | z (mm) |
| --- | --- | --- | --- | --- | --- | --- | --- | --- |
| 245434 | < .001 | < .001 | 75.40 | 7.48 | < .001 | -3 | -15 | -3 |
| 55 | 0.515 | 0.135 | 17.01 | 3.80 | < .001 | -1.5 | -52.5 | -61.5 |

**Table S6.** Peak coordinates where the effect of age at scan on white matter volume is maximum.

| Cluster size (voxels) | FWE corrected p | FDR corrected p | F | equivZ | uncorrected p | x (mm) | y (mm) | z (mm) |
| --- | --- | --- | --- | --- | --- | --- | --- | --- |
| 4170 | < .001 | < .001 | 52.0 | 6.4 | < .001 | 18 | -19.5 | 6 |
| 2344 | < .001 | < .001 | 45.5 | 6.0 | < .001 | -16.5 | -19.5 | 7.5 |
| 1405 | .001 | .004 | 34.9 | 5.4 | < .001 | -12 | -45 | -51 |
| 534 | .001 | .004 | 33.6 | 5.3 | < .001 | -40.5 | -84 | 0 |
| 507 | .035 | .067 | 23.4 | 4.4 | < .001 | -13.5 | 64.5 | -3 |
| 384 | .044 | .076 | 22.7 | 4.4 | < .001 | -15 | -88.5 | 33 |
| 126 | .100 | .145 | 20.5 | 4.2 | < .001 | -13.5 | -37.5 | -21 |
| 449 | .115 | .160 | 20.1 | 4.1 | < .001 | -15 | 55.5 | 27 |
| 310 | .118 | .160 | 20.0 | 4.1 | < .001 | 45 | -78 | 1.5 |
| 62 | .198 | .247 | 18.5 | 4.0 | < .001 | -27 | -52.5 | 46.5 |
| 350 | .212 | .250 | 18.3 | 3.9 | < .001 | -37.5 | -58.5 | -42 |
| 97 | .296 | .324 | 17.3 | 3.8 | < .001 | 0 | -12 | -27 |
| 149 | .330 | .338 | 16.9 | 3.8 | < .001 | 13.5 | 66 | -1.5 |
| 30 | .404 | .357 | 16.3 | 3.7 | < .001 | 25.5 | -34.5 | -30 |
| 84 | .446 | .377 | 15.9 | 3.7 | < .001 | 9 | -51 | 48 |
| 74 | .462 | .389 | 15.8 | 3.7 | < .001 | 45 | 39 | 6 |
| 188 | .488 | .405 | 15.6 | 3.6 | < .001 | 21 | -87 | 24 |
| 126 | .538 | .444 | 15.2 | 3.6 | < .001 | 27 | -96 | -3 |
| 26 | .564 | .463 | 15.0 | 3.6 | < .001 | 18 | 61.5 | 16.5 |
| 30 | .863 | .800 | 12.7 | 3.3 | .001 | 13.5 | 43.5 | 7.5 |

**Table S7.** Peak coordinates where the effect of diagnosis on grey matter volume is maximum.

| Cluster size (voxels) | FWE corrected p | FDR corrected p | F | equivZ | uncorrected p | x (mm) | y (mm) | z (mm) |
| --- | --- | --- | --- | --- | --- | --- | --- | --- |
| 21156 | < .001 | .005 | 13.81 | 5.77 | < .001 | -6 | 31.5 | 55.5 |
| 9629 | .001 | .007 | 12.33 | 5.44 | < .001 | -34.5 | -54 | 51 |
| 205 | .059 | .082 | 8.86 | 4.51 | < .001 | 28.5 | -84 | 1.5 |
| 566 | .063 | .083 | 8.81 | 4.50 | < .001 | 12 | -4.5 | 15 |
| 2701 | .076 | .093 | 8.66 | 4.45 | < .001 | -46.5 | -22.5 | -1.5 |
| 147 | .106 | .106 | 8.38 | 4.37 | < .001 | -16.5 | -4.5 | 12 |
| 1717 | .155 | .114 | 8.05 | 4.26 | < .001 | 43.5 | 19.5 | -1.5 |
| 107 | .174 | .118 | 7.95 | 4.23 | < .001 | -34.5 | -76.5 | 18 |
| 1198 | .225 | .128 | 7.72 | 4.15 | < .001 | 48 | -27 | 34.5 |
| 252 | .380 | .187 | 7.21 | 3.98 | < .001 | -34.5 | 49.5 | 10.5 |
| 87 | .419 | .204 | 7.11 | 3.94 | < .001 | 19.5 | -64.5 | 40.5 |
| 625 | .485 | .239 | 6.95 | 3.88 | < .001 | 10.5 | -6 | 37.5 |
| 138 | .531 | .255 | 6.84 | 3.85 | < .001 | 63 | -28.5 | -7.5 |
| 330 | .553 | .263 | 6.79 | 3.83 | < .001 | -36 | -57 | -13.5 |
| 38 | .611 | .290 | 6.67 | 3.78 | < .001 | 13.5 | -85.5 | 12 |
| 124 | .695 | .334 | 6.48 | 3.71 | < .001 | -48 | 34.5 | 15 |
| 211 | .748 | .365 | 6.36 | 3.67 | < .001 | -28.5 | -79.5 | 33 |
| 88 | .760 | .374 | 6.33 | 3.65 | < .001 | 45 | 39 | 13.5 |
| 72 | .833 | .436 | 6.15 | 3.58 | < .001 | 55.5 | -52.5 | -1.5 |
| 61 | .877 | .478 | 6.02 | 3.53 | < .001 | -31.5 | 54 | -1.5 |
| 46 | .884 | .478 | 6.00 | 3.52 | < .001 | -30 | -18 | 60 |
| 43 | .912 | .518 | 5.90 | 3.48 | < .001 | 51 | 48 | -1.5 |
| 116 | .917 | .528 | 5.88 | 3.47 | < .001 | 42 | -4.5 | -15 |
| 33 | .953 | .582 | 5.71 | 3.41 | < .001 | -10.5 | 61.5 | -1.5 |
| 109 | .957 | .582 | 5.69 | 3.40 | < .001 | 52.5 | 12 | 25.5 |
| 28 | .964 | .603 | 5.64 | 3.38 | < .001 | 10.5 | -72 | 31.5 |
| 45 | .969 | .626 | 5.60 | 3.36 | < .001 | 24 | -81 | 27 |

**Table S8.** Peak coordinates where the effect of diagnosis on white matter volume is maximum.

| Cluster size (voxels) | FWE corrected p | FDR corrected p | F | equivZ | uncorrected p | x (mm) | y (mm) | z (mm) |
| --- | --- | --- | --- | --- | --- | --- | --- | --- |
| 381 | .019 | .070 | 9.34 | 4.66 | < .001 | 19.5 | -99 | 7.5 |
| 265 | .027 | .070 | 9.04 | 4.57 | < .001 | -4.5 | -49.5 | -42 |
| 112 | .028 | .070 | 9.02 | 4.56 | < .001 | -12 | -40.5 | -21 |
| 158 | .050 | .074 | 8.54 | 4.42 | < .001 | -9 | -99 | 4.5 |
| 380 | .154 | .184 | 7.61 | 4.11 | < .001 | 36 | -66 | -40.5 |
| 53 | .163 | .184 | 7.56 | 4.10 | < .001 | 9 | 66 | -1.5 |
| 83 | .311 | .284 | 6.97 | 3.89 | < .001 | 10.5 | -46.5 | -54 |
| 103 | .435 | .314 | 6.63 | 3.77 | < .001 | -28.5 | -4.5 | -6 |
| 33 | .478 | .314 | 6.52 | 3.73 | < .001 | 6 | -52.5 | -37.5 |
| 131 | .502 | .318 | 6.47 | 3.71 | < .001 | 28.5 | -6 | -6 |
| 74 | .621 | .417 | 6.20 | 3.60 | < .001 | -9 | 64.5 | -6 |

**Table S9.** Peak coordinates where the effect of handedness on grey matter volume is maximum.

| Cluster size (voxels) | FWE corrected p | FDR corrected p | F | equivZ | uncorrected p | x (mm) | y (mm) | z (mm) |
| --- | --- | --- | --- | --- | --- | --- | --- | --- |
| 62 | .568 | .998 | 10.19 | 3.75 | < .001 | -9 | -21 | 60 |
| 36 | .890 | .998 | 8.80 | 3.45 | < .001 | 19.5 | 7.5 | -45 |

**Table S10.** Peak coordinates where the effect of handedness on white matter volume is maximum.

| Cluster size (voxels) | FWE corrected p | FDR corrected p | F | equivZ | uncorrected p | x (mm) | y (mm) | z (mm) |
| --- | --- | --- | --- | --- | --- | --- | --- | --- |
| 69 | .290 | .275 | 10.77 | 3.88 | < .001 | 46.5 | -46.5 | 34.5 |

**Table S11.** Peak coordinates where the effect of MRI scan acquisition protocol on grey matter volume is maximum.

| Cluster size (voxels) | FWE corrected p | FDR corrected p | F | equivZ | uncorrected p | x (mm) | y (mm) | z (mm) |
| --- | --- | --- | --- | --- | --- | --- | --- | --- |
| 204409 | < .001 | < .001 | 26.96 | 65535.00 | < .001 | 19.5 | -3 | -6 |
| 528 | < .001 | < .001 | 11.66 | 6.42 | < .001 | 51 | -30 | -28.5 |
| 82 | .141 | .027 | 5.97 | 4.34 | < .001 | 15 | -100.5 | 4.5 |
| 505 | .142 | .028 | 5.97 | 4.34 | < .001 | 49.5 | 3 | -48 |
| 375 | .150 | .029 | 5.94 | 4.32 | < .001 | -34.5 | 18 | -45 |
| 143 | .278 | .054 | 5.58 | 4.14 | < .001 | -46.5 | -4.5 | -49.5 |
| 148 | .561 | .120 | 5.09 | 3.88 | < .001 | -6 | -64.5 | -43.5 |
| 47 | .607 | .136 | 5.02 | 3.84 | < .001 | 24 | -97.5 | 18 |
| 52 | .866 | .273 | 4.62 | 3.61 | < .001 | -43.5 | -60 | 3 |
| 55 | .901 | .311 | 4.54 | 3.56 | < .001 | 22.5 | -85.5 | 42 |
| 43 | .957 | .408 | 4.38 | 3.46 | < .001 | 64.5 | -7.5 | 37.5 |

**Table S12.** Peak coordinates where the effect of MRI scan acquisition protocol on white matter volume is maximum.

| Cluster size (voxels) | FWE corrected p | FDR corrected p | F | equivZ | uncorrected p | x (mm) | y (mm) | z (mm) |
| --- | --- | --- | --- | --- | --- | --- | --- | --- |
| 53604 | < .001 | < .001 | 27.50 | 65535.00 | < .001 | 4.5 | -64.5 | -24 |
| 9838 | < .001 | < .001 | 24.51 | 65535.00 | < .001 | 15 | -97.5 | 7.5 |
| 2171 | < .001 | < .001 | 16.05 | 7.49 | < .001 | -16.5 | 61.5 | -7.5 |
| 235 | < .001 | < .001 | 15.06 | 7.28 | < .001 | -9 | 24 | -22.5 |
| 5588 | < .001 | < .001 | 13.08 | 6.80 | < .001 | 39 | -16.5 | 51 |
| 111 | < .001 | < .001 | 11.47 | 6.37 | < .001 | -51 | -30 | -25.5 |
| 360 | < .001 | < .001 | 11.02 | 6.24 | < .001 | 49.5 | -30 | -24 |
| 339 | < .001 | < .001 | 1.70 | 6.14 | < .001 | 1.5 | 25.5 | -22.5 |
| 634 | < .001 | < .001 | 8.80 | 5.51 | < .001 | 12 | 66 | -4.5 |
| 1020 | .008 | .003 | 7.27 | 4.92 | < .001 | 46.5 | 39 | 4.5 |
| 839 | .010 | .003 | 7.10 | 4.85 | < .001 | 1.5 | -15 | -25.5 |
| 106 | .021 | .007 | 6.74 | 4.70 | < .001 | -4.5 | 43.5 | 3 |
| 363 | .021 | .007 | 6.73 | 4.69 | < .001 | 42 | -72 | 28.5 |
| 1367 | .029 | .009 | 6.57 | 4.62 | < .001 | -9 | 13.5 | 4.5 |
| 56 | .035 | .011 | 6.46 | 4.57 | < .001 | -36 | -90 | -6 |
| 182 | .043 | .013 | 6.35 | 4.52 | < .001 | 7.5 | -4.5 | 37.5 |
| 66 | .078 | .023 | 6.03 | 4.37 | < .001 | -37.5 | -85.5 | 7.5 |
| 30 | .149 | .044 | 5.68 | 4.19 | < .001 | -31.5 | -81 | 27 |
| 120 | .260 | .076 | 5.35 | 4.02 | < .001 | 55.5 | -36 | 25.5 |
| 28 | .276 | .081 | 5.32 | 4.00 | < .001 | -28.5 | -78 | -16.5 |
| 46 | .497 | .163 | 4.92 | 3.78 | < .001 | -42 | -67.5 | -9 |
| 50 | .523 | .174 | 4.88 | 3.76 | < .001 | -13.5 | 4.5 | 19.5 |
| 29 | .555 | .187 | 4.84 | 3.73 | < .001 | -39 | -72 | 36 |
| 57 | .560 | .189 | 4.83 | 3.73 | < .001 | 9 | 13.5 | 43.5 |
| 60 | .796 | .340 | 4.48 | 3.52 | < .001 | 9 | -61.5 | 25.5 |
| 29 | .917 | .500 | 4.24 | 3.37 | < .001 | 0 | 15 | 16.5 |
| 26 | .963 | .639 | 4.09 | 3.27 | .001 | 28.5 | 21 | 33 |

**Table S13.** Peak coordinates where the effect of sex on grey matter volume is maximum.

| Cluster size (voxels) | FWE corrected p | FDR corrected p | F | equivZ | uncorrected p | x (mm) | y (mm) | z (mm) |
| --- | --- | --- | --- | --- | --- | --- | --- | --- |
| 118588 | < .001 | < .001 | 57.59 | 6.70 | < .001 | 21 | -6 | -28.5 |
| 10597 | < .001 | < .001 | 39.27 | 5.67 | < .001 | 9 | 25.5 | 66 |
| 1651 | .011 | .009 | 28.25 | 4.87 | < .001 | 48 | -58.5 | 31.5 |
| 380 | .117 | .052 | 21.57 | 4.28 | < .001 | -45 | -54 | 36 |
| 376 | .313 | .130 | 18.63 | 3.98 | < .001 | 25.5 | 61.5 | 27 |
| 160 | .322 | .133 | 18.54 | 3.97 | < .001 | 28.5 | -76.5 | 36 |
| 889 | .396 | .155 | 17.85 | 3.89 | < .001 | -15 | 57 | 13.5 |
| 504 | .397 | .155 | 17.84 | 3.89 | < .001 | -45 | -16.5 | 58.5 |
| 168 | .467 | .182 | 17.26 | 3.83 | < .001 | 19.5 | 54 | 39 |
| 288 | .493 | .191 | 17.06 | 3.81 | < .001 | -57 | 25.5 | 3 |
| 377 | .523 | .204 | 16.83 | 3.78 | < .001 | -55.5 | -1.5 | 33 |
| 161 | .527 | .204 | 16.80 | 3.78 | < .001 | 25.5 | -90 | 33 |
| 128 | .638 | .252 | 15.99 | 3.68 | < .001 | -43.5 | -37.5 | 55.5 |
| 65 | .724 | .303 | 15.37 | 3.61 | < .001 | 49.5 | 48 | 12 |
| 91 | .781 | .344 | 14.93 | 3.56 | < .001 | 22.5 | -51 | 72 |
| 84 | .883 | .442 | 14.01 | 3.44 | < .001 | 34.5 | -7.5 | 57 |
| 45 | .886 | .444 | 13.98 | 3.44 | < .001 | 19.5 | -37.5 | -3 |
| 79 | .886 | .444 | 13.98 | 3.44 | < .001 | 21 | -72 | 57 |
| 44 | .895 | .453 | 13.88 | 3.42 | < .001 | -49.5 | 7.5 | 42 |
| 28 | .907 | .472 | 13.74 | 3.41 | < .001 | 27 | -51 | 58.5 |
| 25 | .942 | .549 | 13.26 | 3.34 | < .001 | 36 | -93 | 6 |
| 60 | .947 | .560 | 13.18 | 3.33 | < .001 | 16.5 | -30 | 75 |

**Table S14.** Peak coordinates where the effect of sex on white matter volume is maximum.

| Cluster size (voxels) | FWE corrected p | FDR corrected p | F | equivZ | uncorrected p | x (mm) | y (mm) | z (mm) |
| --- | --- | --- | --- | --- | --- | --- | --- | --- |
| 196773 | < .001 | < .001 | 74.96 | 7.46 | < .001 | 9 | -9 | -15 |
| 59 | .342 | .123 | 17.27 | 3.83 | < .001 | -52.5 | -3 | 42 |
|  |  |  |  |  |  |  |  |  |

**Table S15.** Association analyses between age at scan, sex and handedness and each SNP’s proxy genotype.

| Gene/SNP**/Proxy**  (statistical model - risk genotype on the left) | **Age at scan (years)** | **Sex (M/F)** | **Handedness (R/L/A)** |
| --- | --- | --- | --- |
| DISC1 rs11122319  **rs1417585**  (CC *vs.* CT *vs.* TT) | F = .06, p = .940 | χ^2^ = 1.08, p = .582 | χ^2^ = 5.88, p = .208  (Fisher’s exact test: p = .193) |
| **CC** (n=31) | 32.9 ± 13.7 | 20/11 | 29/0/2 |
| **CT** (n=61) | 32.4 ± 13.1 | 36/25 | 54/6/1 |
| **TT** (n=21) | 31.6 ± 12.8 | 15/6 | 20/1/0 |
| DISC1 rs2793092  **rs2793098**  (AA *vs.* G-car.) | T = 0.34, p = .732 | χ^2^=1.79, p=.181 | χ^2^=0.39, p=.823  (Fisher’s exact test: p=.869) |
| **AA** (n=69) | 32.7 ± 13.3 | 40/29 | 62/5/2 |
| **AG+GG** (n=44) | 31.9 ± 13.0 | 31/13 | 41/2/1 |
| DISC1 rs2793092  **rs2793098**  (AA *vs.* AG *vs.* GG) | F = .08, p = .919 | χ^2^ = 2.01, p = .366  (Fisher’s exact test: p = .427) | χ^2^ = 2.78, p = .595  (Fisher’s exact test: p = .403) |
| **AA** (n=69) | 32.7 ± 13.3 | 40/29 | 62/5/2 |
| **AG** (n=39) | 32.04 ± 13.0 | 27/12 | 37/1/1 |
| **GG** (n=5) | 30.6 ± 13.9 | 4/1 | 4/1/0 |
| ZNF804A rs1344706  **rs11681373**^1^  (AA *vs.* G-car.) | T = 0.61, p = .545 | χ^2^ = 0.11 p = .738 | χ^2^ = 3.82, p = .148  (Fisher’s exact test: p = .166) |
| **AA** (n=46) | 33.1 ± 14.0 | 30/16 | 39/5/2 |
| **AG + GG** (n=66) | 31.6 ± 12.2 | 41/25 | 63/2/1 |
| ZNF804A rs1344706  **rs11681373**^1^  (A-car. *vs.* GG) | T = 1.78, p = .078 | χ^2^ = 3.32 p = .069 | χ^2^ = 2.10, p = .349  (Fisher’s exact test: p = .763) |
| **AA+AG** (n=94) | 31.2 ± 13.4 | 63/31 | 84/7/3 |
| **GG** (n=18) | 37.1 ± 15.1 | 8/10 | 18/0/0 |
| ZNF804A rs1344706  **rs11681373**^1^  (AA *vs.* AG *vs.* GG) | F = 2.52, p = .085 | χ^2^ = 3.45 p = .179 | χ^2^ = 4.45, p = .349  (Fisher’s exact test: p = .521) |
| **AA** (n=46) | 33.1 ± 14.0 | 30/16 | 39/5/2 |
| **AG** (n=48) | 29.5 ± 10.4 | 33/15 | 45/2/1 |
| **GG** (n=18) | 37.1 ± 15.1 | 8/10 | 18/0/0 |
| NRG1 rs35753505  (C-car. *vs.* TT) | T = 3.28, p = .001*** | χ^2^ = .01, p = .934 | χ^2^ = 1.29, p = .524  (Fisher’s exact test: p = .580) |
| **CC+CT** (n=64) | 35.8 ± 14.3 | 40/24 | 58/5/1 |
| **TT** (n=49) | 28.0 ± 9.9 | 31/18 | 45/2/2 |
| NRG1 rs6994992  **rs4733264**  (G-car. *vs.* CC) | T = 2.51, p = .014* | χ^2^ = .00, p = .969 | χ^2^ = 0.54, p = .763  (Fisher’s exact test: p = .869) |
| **GG+GC** (n=67) | 34.9 ± 14.0 | 42/25 | 60/5/2 |
| **CC** (n=46) | 28.8 ± 10.8 | 29/17 | 43/2/1 |
| NRG1 rs6994992  **rs4733264**  (GG *vs.* GC *vs.* CC) | F = 3.34, p = .039* | χ^2^ = 4.76 p = .093 | χ^2^ = 1.57, p = .815  (Fisher’s exact test: p = .692) |
| **GG** (n=15) | 36.8 ± 15.5 | 13/2 | 13/1/1 |
| **GC** (n=52) | 34.4 ± 13.7 | 29/23 | 47/4/1 |
| **CC** (n=46) | 28.8 ± 10.8 | 29/17 | 43/2/1 |
| BDNF rs6265  **rs4923457**  (T-car. *vs.* AA) | T = 0.42, p = .674 | χ^2^ = 0.48, p = .487 | χ^2^ = 0.60, p = .740  (Fisher’s exact test: p = .633) |
| **TT+TA** (n=34) | 33.2 ± 14.0 | 23/11 | 30/3/1 |
| **AA** (n=79) | 32.1 ± 12.8 | 48/31 | 73/4/2 |
| BDNF rs6265  **rs4923457**  (TA *vs.* AA) | T = .492, p = .624 | χ^2^ = 0.61, p = .434 | χ^2^ = 1.07, p = .587  (Fisher’s exact test: p = .493) |
| **TA** (n=29) | 33.5 ± 14.4 | 20/9 | 25/3/1 |
| **AA** (n=79) | 32.1 ± 12.8 | 48/31 | 73/4/2 |
| CACNA1C rs1006737  **rs769087**  (A-car. *vs.* GG) | T = 0.61, p = .541 | χ^2^ = .38, p = .540 | χ^2^ = 0.12, p = .942  (Fisher’s exact test: p = 1) |
| **AA+AG** (n=74) | 33.0 ± 13.8 | 45/29 | 67/5/2 |
| **GG** (n=39) | 31.4 ± 11.7 | 26/13 | 36/2/1 |
| CACNA1C rs1006737  **rs769087**  (AA *vs.* AG *vs.* GG) | F = 0.30, p = .745 | χ^2^ = 1.99 p = .370 | χ^2^ = 0.65, p = .957  (Fisher’s exact test: p > .999) |
| **AA** (n=15) | 34.4 ± 13.8 | 7/8 | 14/1/0 |
| **AG** (n=59) | 32.6 ± 13.9 | 38/21 | 53/4/2 |
| **GG** (n=39) | 31.4 ± 11.7 | 26/13 | 36/2/1 |

^1^Missing genotype for 1 subject.

**Table S16.** Association analysis between subject’s diagnosis and genotype.

| Gene/SNP/**Proxy**  (risk genotype on the left) | **Schizophrenia** | **Bipolar Disorder** | **Controls** | **ARMS-NT** | **ARMS-T** |
| --- | --- | --- | --- | --- | --- |
| DISC1 rs11122319  **rs1417585**  (CC *vs.*CT *vs.* TT) | χ^2^ = 13.09, p = .109 (Fisher’s exact test: p = .124) | | | | |
| **CC** (n=31) | 2 | 7 | 12 | 10 | 0 |
| **CT** (n=61) | 6 | 12 | 25 | 12 | 6 |
| **TT** (n=21) | 6 | 2 | 7 | 3 | 3 |
| DISC1 rs2793092  **rs2793098**  (AA *vs.* G-car.) | χ^2^ = 2.22, p = .695 (Fisher’s exact test: p = .708) | | | | |
| **AA** (n=69) | 9 | 10 | 29 | 15 | 6 |
| **AG+GG** (n=44) | 5 | 11 | 15 | 10 | 3 |
| DISC1 rs2793092  **rs2793098**  (AA *vs.* AG *vs.* GG) | χ^2^ = 5.26, p = .730 (Fisher’s exact test: p = .722) | | | |  |
| **AA** (n=69) | 9 | 10 | 29 | 15 | 6 |
| **AG** (n=39) | 4 | 10 | 12 | 10 | 3 |
| **GG** (n=5) | 1 | 1 | 3 | 0 | 0 |
| ZNF804A rs1344706  **rs11681373**^1^  (AA *vs.* G-car.) | χ^2^ = 6.32, p = .177 (Fisher’s exact test: p = .163) | | | |  |
| **AA** (n=46) | 5 | 4 | 22 | 11 | 4 |
| **AG + GG** (n=66) | 9 | 17 | 21 | 14 | 5 |
| ZNF804A rs1344706  **rs11681373**^1^  (A-car. *vs.* GG) | χ^2^ = 4.64, p = .326 (Fisher’s exact test: p = .272) | | | | |
| **AA+AG** (n=94) | 10 | 16 | 38 | 23 | 7 |
| **GG** (n=18) | 4 | 5 | 5 | 2 | 2 |
| ZNF804A rs1344706  **rs11681373**^1^  (AA *vs.* AG *vs.* GG) | χ^2^ = 9.45, p = .306 (Fisher’s exact test: p = .254) | | | | |
| **AA** (n=46) | 5 | 4 | 22 | 11 | 4 |
| **AG** (n=48) | 5 | 12 | 16 | 12 | 3 |
| **GG** (n=18) | 4 | 5 | 5 | 2 | 2 |
| NRG1 rs35753505  (C-car. *vs.* TT) | χ^2^ = 5.10, p = .277 (Fisher’s exact test: p = .283) | | | |  |
| **CC+CT** (n=64) | 10 | 11 | 28 | 10 | 5 |
| **TT** (n=49) | 4 | 10 | 16 | 15 | 4 |
| NRG1 rs6994992  **rs4733264**  (G-car. *vs.* CC) | χ^2^ = 1.95, p = .744 (Fisher’s exact test: p = .753) | | | |  |
| **GG+GC** (n=67) | 8 | 13 | 28 | 12 | 6 |
| **CC** (n=46) | 6 | 8 | 16 | 13 | 3 |
| NRG1 rs6994992  **rs4733264**  (GG *vs.* GC *vs.* CC) | χ^2^ = 5.04, p = .753 (Fisher’s exact test: p = .831) | | | | |
| **GG** (n=15) | 1 | 4 | 7 | 3 | 0 |
| **GC** (n=52) | 7 | 9 | 21 | 9 | 6 |
| **CC** (n=46) | 6 | 8 | 16 | 13 | 3 |
| BDNF rs6265  **rs4923457**  (T-car. *vs.* AA) | χ^2^ = 2.17, p = .704 (Fisher’s exact test: p = .719) | | | |  |
| **TT+TA** (n=34) | 5 | 4 | 14 | 9 | 2 |
| **AA** (n=79) | 9 | 17 | 30 | 16 | 7 |
| BDNF rs6265  **rs4923457**  (TA *vs.* AA) | χ^2^ = 2.21, p = .717 (Fisher’s exact test: p = .757) | | | | |
| **TA** (n=29) | 3 | 4 | 14 | 7 | 1 |
| **AA** (n=79) | 9 | 17 | 30 | 16 | 7 |
| CACNA1C rs1006737  **rs769087**  (A-car. *vs.* GG) | χ^2^ = 1.34, p = .855 (Fisher’s exact test: p = .878) | | | |  |
| **AA+AG** (n=74) | 9 | 15 | 28 | 15 | 7 |
| **GG** (n=39) | 5 | 6 | 16 | 10 | 2 |
| CACNA1C rs1006737  **rs769087**  (AA *vs.* AG *vs.* GG) | χ^2^ = 2.91, p = .940 (Fisher’s exact test: p = .936) | | | | |
| **AA** (n=15) | 2 | 4 | 4 | 4 | 1 |
| **AG** (n=59) | 7 | 11 | 24 | 11 | 6 |
| **GG** (n=39) | 5 | 6 | 16 | 10 | 2 |

^1^Missing genotype for 1 subject.

**Table S17.** Association analysis between MRI scanning protocol and genotype.

| **Gene/**SNP**/**Proxy  (risk genotype on the left) | **P1** | **P2** | **P3** | **P4** | **P5** | **P6** | **P7** | **P8** |
| --- | --- | --- | --- | --- | --- | --- | --- | --- |
| DISC1 rs11122319  **rs1417585**  (CC *vs.* CT *vs.* TT) | χ^2^ = 9.15, p = .822 (Fisher’s exact test: p = .796) | | | | | | | |
| **CC** (n=31) | 4 | 3 | 6 | 7 | 0 | 0 | 5 | 6 |
| **CT** (n=61) | 6 | 4 | 12 | 15 | 4 | 4 | 5 | 11 |
| **TT** (n=21) | 3 | 1 | 3 | 4 | 2 | 2 | 4 | 2 |
| DISC1 rs11122319  **rs2793098**  (AA *vs.* G-car.) | χ^2^ = 3.76, p = .807 (Fisher’s exact test: p = .810) | | | | | | | |
| **AA** (n=69) | 9 | 4 | 11 | 18 | 4 | 3 | 10 | 10 |
| **AG+GG** (n=44) | 4 | 4 | 10 | 8 | 2 | 3 | 4 | 9 |
| DISC1 rs11122319  **rs2793098**  (AA *vs.* AG *vs.* GG) | χ^2^ = 11.50, p = .658 (Fisher’s exact test: p = .633) | | | | | | | |
| **AA** (n=69) | 9 | 4 | 11 | 18 | 4 | 3 | 10 | 10 |
| **AG** (n=39) | 3 | 4 | 9 | 7 | 2 | 3 | 2 | 9 |
| **GG** (n=5) | 1 | 0 | 1 | 1 | 0 | 0 | 2 | 0 |
| ZNF804A rs1344706  **rs11681373**^1^  (AA *vs.* G-car.) | χ^2^ = 4.86, p = .677 (Fisher’s exact test: p = .687) | | | | | | | |
| **AA** (n=46) | 6 | 1 | 9 | 10 | 4 | 3 | 6 | 7 |
| **AG + GG** (n=66) | 7 | 7 | 12 | 15 | 2 | 3 | 8 | 12 |
| ZNF804A rs1344706  **rs11681373**^1^  (A-car. *vs.* GG) | χ^2^ = 17.29, p = .016* (Fisher’s exact test: p = .026*) | | | | | | | |
| **AA+AG** (n=94) | 13 | 3 | 18 | 20 | 5 | 5 | 12 | 18 |
| **GG** (n=18) | 0 | 5 | 3 | 5 | 1 | 1 | 2 | 1 |
| ZNF804A rs1344706  **rs11681373**^1^  (AA *vs.* AG *vs.* GG) | χ^2^ = 20.47, p = .116 (Fisher’s exact test: p = .230) | | | | | | | |
| **AA** (n=46) | 6 | 1 | 9 | 10 | 4 | 3 | 6 | 7 |
| **AG** (n=48) | 7 | 2 | 9 | 10 | 1 | 2 | 6 | 11 |
| **GG** (n=18) | 0 | 5 | 3 | 5 | 1 | 1 | 2 | 1 |
| NRG1 rs35753505  (C-car. *vs.* TT) | χ^2^ = 10.65, p = .155 (Fisher’s exact test: p = .164) | | | | | | | |
| **CC+CT** (n=64) | 10 | 6 | 14 | 12 | 4 | 4 | 8 | 6 |
| **TT** (n=49) | 3 | 2 | 7 | 14 | 2 | 2 | 6 | 13 |
| NRG1 rs6994992  **rs4733264**  (G-car. *vs.* CC) | χ^2^ = 8.13, p = .322 (Fisher’s exact test: p = .310) | | | | | | | |
| **GG+GC** (n=67) | 11 | 5 | 15 | 12 | 3 | 4 | 8 | 9 |
| **CC** (n=46) | 2 | 3 | 6 | 14 | 3 | 2 | 6 | 10 |
| NRG1 rs6994992  **rs4733264**  (GG *vs.* GC *vs.* CC) | χ^2^ = 14.35, p = .424 (Fisher’s exact test: p = .332) | | | | | | | |
| **GG** (n=15) | 2 | 2 | 4 | 3 | 1 | 2 | 0 | 1 |
| **GC** (n=52) | 9 | 3 | 11 | 9 | 2 | 2 | 8 | 8 |
| **CC** (n=46) | 2 | 3 | 6 | 14 | 3 | 2 | 6 | 10 |
| BDNF rs6265  **rs4923457**  (T-car. *vs.* AA) | χ^2^ = 5.92, p = .549 (Fisher’s exact test: p = .586) | | | | | | | |
| **TT+TA** (n=34) | 6 | 1 | 4 | 9 | 1 | 3 | 4 | 6 |
| **AA** (n=79) | 7 | 7 | 17 | 17 | 5 | 3 | 10 | 13 |
| BDNF rs6265  **rs4923457**  (TA *vs.* AA) | χ^2^ = 8.53, p = .292 (Fisher’s exact test: p = .304) | | | | | | | |
| **TA** (n=29) | 6 | 0 | 4 | 8 | 1 | 3 | 4 | 3 |
| **AA** (n=79) | 7 | 7 | 17 | 17 | 5 | 3 | 10 | 13 |
| CACNA1C rs1006737  **rs769087**  (A-car. *vs.* GG) | χ^2^ = 7.13, p = .415 (Fisher’s exact test: p = .420) | | | | | | | |
| **AA+AG** (n=74) | 7 | 4 | 12 | 21 | 5 | 5 | 8 | 12 |
| **GG** (n=39) | 6 | 4 | 9 | 5 | 1 | 1 | 6 | 7 |
| CACNA1C rs1006737  **rs769087**  (AA *vs.* AG *vs.* GG) | χ^2^ = 8.89, p = .838 (Fisher’s exact test: p = .810) | | | | | | | |
| **AA** (n=15) | 1 | 1 | 2 | 4 | 1 | 1 | 1 | 4 |
| **AG** (n=59) | 6 | 3 | 10 | 17 | 4 | 4 | 7 | 8 |
| **GG** (n=39) | 6 | 4 | 9 | 5 | 1 | 1 | 6 | 7 |

^1^Missing genotype for 1 subject.

**Table S18.** Association analysis between total intracranial volume and genotype.

| Gene**/**SNP**/Proxy**  **(**risk genotype on the left) | **Total intracranial volume** (cm^3^) |
| --- | --- |
| DISC1 rs11122319  **rs1417585**  (CC *vs.* CT *vs.* TT) | F = 0.35, p = .709 |
| **CC** (n=31) | 1555 ± 132 |
| **CT** (n=61) | 1533 ± 132 |
| **TT** (n=21) | 1530 ± 130 |
| DISC1 rs11122319  **rs2793098**  (AA *vs.* G-car.) | T = 0.48, p = .632 |
| **AA** (n=69) | 1543 ± 126 |
| **AG+GG** (n=44) | 1531 ± 140 |
| DISC1 rs11122319  **rs2793098**  (AA *vs.* AG *vs.* GG) | F = 0.13, p = .874 |
| **AA** (n=69) | 1543 +/- 126 |
| **AG** (n=5) | 1520 ± 127 |
| **GG** (n=39) | 1532 ± 143 |
| ZNF804A rs1344706  **rs11681373**^1^  (AA *vs.* G-car.) | T = 0.20, p = .839 |
| **AA** (n=46) | 1537 ± 132 |
| **AG + GG** (n=66) | 1542 ± 129 |
| ZNF804A rs1344706  **rs11681373**^1^  (A-car. *vs.* GG) | T = 1.47, p = .144 |
| **AA+AG** (n=94) | 1548 ± 127 |
| **GG** (n=18) | 1499 ± 139 |
| ZNF804A rs1344706  **rs11681373**^1^  (AA *vs.* AG *vs.* GG) | F = 1.40, p = .252 |
| **AA** (n=46) | 1537 ± 132 |
| **AG** (n=48) | 1559 ± 123 |
| **GG** (n=18) | 1499 ± 139 |
| NRG1 rs35753505  (C-car. *vs.* TT) | T = 1.63, p = .106 |
| **CC+CT** (n=64) | 1521 ± 125 |
| **TT** (n=49) | 1561 ± 136 |
| NRG1 rs6994992  **rs4733264**  (G-car. *vs.* CC) | T = 0.67, p = .506 |
| **GG+GC** (n=67) | 1531 ± 126 |
| **CC** (n=46) | 1548 ± 139 |
| NRG1 rs6994992  **rs4733264**  (GG *vs.* GC *vs.* CC) | F = 0.45, p = .783 |
| **GG** (n=15) | 1538 ± 110 |
| **GC** (n=52) | 1529 ± 131 |
| **CC** (n=46) | 1548 ± 139 |
| BDNF rs6265  **rs4923457**  (T-car. *vs.* AA) | T = 0.68, p = .499 |
| **TT+TA** (n=34) | 1551 ± 130 |
| **AA** (n=79) | 1533 ± 132 |
| BDNF rs6265  **rs4923457**  (TA *vs.* AA) | T = 0.48, p = .632 |
| **TA** (n=29) | 1546 ± 129 |
| **AA** (n=79) | 1533 ± 132 |
| CACNA1C rs1006737  **rs769087**  (A-car. *vs.* GG) | T = 0.01, p = .990 |
| **AA+AG** (n=74) | 1538 ± 120 |
| **GG** (n=39) | 1538 ± 152 |
| CACNA1C rs1006737  **rs769087**  (AA *vs.* AG *vs.* GG) | F = 0.07, p = .936 |
| **AA** (n=15) | 1527 ± 140 |
| **AG** (n=59) | 1541 ± 115 |
| **GG** (n=39) | 1538 ± 152 |

^1^Missing genotype for 1 subject.

**Table S19.** Excluded studies assessing a SNP genotype effect on brain volume, with reasons for exclusion.

| Gene (SNP’s) | Studies excluded and SNPs tested | Reasons for exclusion |
| --- | --- | --- |
| DISC1 | Kahler 2012 (excluded SNPs rs1417584 and rs821589) | SNPs not present in our sample, and neither proxys of it with r^2^ > 0,8 |
|  | Wei 2012b (rs821597) | Genotype main effect non-significant |
|  | Takahashi 2009 (rs821616) | Genotype main effect non-significant after multiple comparisons correction |
|  | Di Giorgio 2008 (rs821616) | Genotype main effect non-significant after multiple comparisons correction |
|  | Trost 2013 (rs6675281 and rs821616) | Genotype main effect not assessed with multiple comparisons correction |
|  | Knickmeyer 2014 (rs821616 and rs6675281) | MRI brain scans of neonates |
|  | Chakravarty 2012 (rs6675281 and rs821616) | Genotype main effect non-significant after multiple comparisons correction |
| ZNF804A | Bergmann 2013 (63 SNPs, including rs1344706) | Genotype main effect non-significant |
|  | Cousijn 2012 (rs1344706) | Genotype main effect non-significant |
| NRG1 | Addington 2007 (420M9‐1395) | Longitudinal study, with childhood-onset schizophrenia subjects and their parents |
|  | Gruber 2008 (HAPice haplotype: (SNP8NRG221533 = rs35753505, 478B14‐848, 420M9‐1395) | Genotype main effect non-significant, for the SNP alone |
|  | Haukvik 2010 (rs2954041) | Genotype main effect non-significant |
|  | Wang 2009 (rs35753505) | Brain effects assessed through DTI only |
|  | Thirunavukkarasu 2014 (rs35753505) | Genotype main effect “trend” only |
|  | Suarez‐Pinilla 2015 (rs35753505) | Longitudinal study, only assessing genotype main effects over time |
|  | Winterer 2008 (rs35753505) | Genotype main effect non-significant |
|  | Tosato 2012 (rs4623364) | SNPs not present in our sample, and neither proxys of it with r^2^ > 0,8 |
|  | Dutt 2009 (s35753505, 478B14‐848, 420M9‐1395) | Genotype main effect non-significant |
|  | Knickmeyer 2014 (rs35753505 and rs6994992) | MRI brain scans of neonates |
|  | Hall 2006 (rs6994992) | Genotype main effect non-significant |
|  | Bousman 2018 (rs4281084 and rs12155594) | Genotype main effect non-significant |
| BDNF | Cao 2016 (rs6265) | Genotype main effect only marginal (p=0.055) |
|  | Agartz 2006 (rs6265; 270 C/T; 633 T/A; 11757 G/C) | Genotype main effect non-significant after multiple comparisons correction |
|  | Dutt 2009 (rs6265) | Genotype main effect non-significant |
|  | Knickmeyer 2014 (rs6265) | MRI brain scans of neonates |
|  | Aas 2013 (rs6265) | Genotype main effect not assessed |
|  | Mirakhur 2009 (rs6265) | Genotype main effect on brain measured through gyrification |
|  | Ho 2007 (rs6265) | Longitudinal study, only assessing genotype main effects over time |
|  | Karnik 2010 (rs6265) | Genotype main effect non-significant |
|  | Takahashi 2008 (rs6265) | Genotype main effect not corrected for multiple comparisons |
|  | Koolschijn 2010 (rs6265) | Genotype main effect non-significant |
|  | Smith 2012 (rs6265) | Genotype main effect non-significant |
|  | Sublette 2008 (rs6265) | Genotype main effect not assessed |
|  | Zeni 2016 (rs6265) | MRI scans of subjects with less then 18 years old |
| CACNA1C | Kempton 2009 (rs1006737) | Genotype main effect not corrected for multiple comparisons |
|  | Soeiro-de-Souza 2012 (rs1006737) | Genotype main effect non-significant |
| ANK3 | Tesli 2013 (rs9804190, rs10994336, rs10994397, rs1938526) | Genotype main effect non-significant after multiple comparisons correction |
|  | Lippard 2016 (rs9804190) | Genotype significant main effect assessed through sMRI not corrected for multiple comparison |
|  | Ota 2016 (rs10761482) | Only age-related significant genotype effect |
| IL-1 beta | Papiol 2008 (rs16944) | Genotype main effect non-significant after multiple comparisons correction |
|  | Meisenzahl 2001 (rs16944) | Genotype main effect non-significant |
|  | Papiol 2005 (rs16944) | Genotype main effect non-significant |
| IL-1RN | Papiol 2005 (86-bp VNTR) | Single study reporting genotype significant main effect for a IL-1RN SNP |
|  | Roiz-Santiáñez 2008 (86-bp VNTR) | Genotype main effect non-significant after multiple comparisons correction |
| APOE e4 allele | Hata 2002 | Genotype main effect non-significant after multiple comparisons correction |
| 5-HTTLPR | Scherk 2009a | Single study reporting genotype significant main effect for a IL-1RN SNP |
|  | Benedetti 2014 (s/l alleles) | Genotype main effect non-significant |
| NRGN | Pohlack 2011 (rs12807809) | Genotype main effect non-significant |
|  | Rose 2011 (rs12807809) | Genotype main effect non-significant |
| CSMD1 | Rose 2013 (rs10503253) | Genotype main effect non-significant |
| GSK-3β | Benedetti 2015 (rs334558) | Genotype main effect non-significant |
| SLC1A2 | Poletti 2014 (181 A to C) | Genotype main effect non-significant |
|  | | |
| Genes with only one validating study: | Excluded study and SNP | |
| DGKH | Kittel-Schneider 2015 (rs994856/rs9525580/rs9525584) | |
| GRIN2B | Kuswanto 2013 (rs890) | |
| MIR137, CCDC68, CNNM2, NT5C2, MMP16, CSMD1, PCGEM1 | Oertel-Knochel 2015 (respectively per gene: rs1625579, rs12966547, rs7914558, rs111915801, rs7004633, rs10503253, rs17662626) | |
| 15q12 | Bakken 2011 (rs4906844, rs11633924) | |
| NRGN | Ohi 2012 (rs12807809) | |
| MHC | Agartz 2011 (rs2596532) | |
| VRK2 | Li 2012 (rs2312147) | |
| TCF4 | Wirgenes 2012 (rs12966547, rs9960767) | |
| CNNM2 | Rose 2014 (rs7914558) | |
| HAPice | Cannon 2012 | |
| PLXNB3 | Rujescu 2007 (V598I, E1156D) | |
| AKT1 | Tan 2008 (rs1130233) | |
| MOG | Cannon 2012 (rs2857766) | |
| DARPP-32 | Meyer-Lindenberg 2007 (M04-03 and M11-15) | |

**Table S20.** Demographics of the present study’s sample.

| **Diagnostic group** | **Age at scan (years)** | **Sex (M/F)** | **Handedness (R/L/A)** |
| --- | --- | --- | --- |
| **Schizophrenia (n=14)** | 37.2 ± 11.3 | 10/4 | 14/0/0 |
| **Bipolar disorder (n=21)** | 39.7 ± 12.7 | 10/11 | 19/0/2 |
| **Control (n=44)** | 34.9 ± 14.4 | 26/18 | 37/6/1 |
| **ARMS-NT (n=25)** | 22.4 ± 3.1 | 19/6 | 24/1/0 |
| **ARMS-T (n=9)** | 23.6 ± 4.9 | 3/6 | 9/0/0 |
| **Group comparison** | F = 9.28, p < .001***^a^ | ꭓ^2^ = 4.70, p = .319  (Fisher’s exact test: p = .332) | ꭓ^2^ = 12.39, p = .135  (Fisher’s exact test: p = .260) |

Footnotes: ^a^ One-way ANOVA; *p<.05; **p<.01; ***p<.001

(Abbreviation, in alphabetical order): ARMS-NT = At Risk Mental State Non-transitioned (to psychosis); ARMS-T = At Risk Mental State Transitioned (to psychosis)

**Table S21.** Sample size, diagnostic and ancestry composition, per included primary study.

| **Gene / SNP** | **Authors / SNP** | **Sample size (per diagnosis/ condition)** | **Sample ancestry (registered as reported by the primary studies)** |
| --- | --- | --- | --- |
| *DISC1* | Kahler et al, 2012 (rs11122319) | 355 (HC: 171; PH: 184) | Caucasians |
|  | Mata et al, 2010 (rs2793092) | 112 (HC: 21; SZ: 91) | Not-specified |
| *ZNF804A*  (rs1344706) | Voineskos et al, 2011 | 62 (HS) | Caucasian |
|  | Donohoe et al, 2011 | 108 (HC: 38; SZ: 70) | Caucasian italian |
|  | Lencz et al, 2010 | 39 (HS) | Caucasian |
|  | Schultz et al., 2014 | 95 (SZ: 50; HC 40) | Caucasian |
|  | Wassink et al, 2012 | 553 (HC:198; SZ spectrum: 335) | Not-specified |
|  | Wei et al, 2012 | 149 (HC: 69; SZ spectrum: 80) | Han chinese |
| *NRG1* | Cannon et al, 2012 (rs35753505) | 189 (HC: 39; UR: 80; SZ/BDI: 70) | “168” Europeans |
|  | Mata et al, 2009 (rs6994992) | 111 (HC: 16; SZ: 95) | Not-specified |
|  | McIntosh et al, 2008 (rs6994992) | 87 (HS) | Scottish |
| *BDNF*  (rs6265) | Ho et al, 2006 | 437 (HC: 144; SZ spectrum: 293) | Not-specified |
|  | Montag et al, 2009 | 87 (HS) | Caucasian, German origin |
|  | Chepenik et al, 2009 | 38 (HC: 18; BD: 20) | “31” European-american; “3” African-american; “4” Other ancestry |
|  | Nemoto et al, 2006 | 130 (HS) | Japanese |
|  | Pezawas et al, 2004 | 214 (HS) | “163” Caucasian; “26” African-American; “14” Hispanic; “8” Asian; “1” Native American |
|  | Yang et al, 2012 | 81 (HS) | Chinese |
|  | Szeszko et al, 2005 | 44 (HC: 25; SZ spectrum: 19) | Caucasians |
|  | Bueller et al, 2006 | 36 (HS) | “4” Caucasian; “7” African-American; “5” Asian |
|  | Yang et al, 2012 | 81 (HS) | Chinese |
|  | Matsuo et al, 2009 | 84 (HC: 42; BD: 42) | Not specified |
| *CACNA1C*  (rs1006737) | Perrier et al, 2011 | 91 (HC: 50; BDI: 41) | White british descent |
|  | Wang et al, 2011 | 55 (HS) | European Americans |
|  | Wolf et al, 2013 | 72 (HC: 16; 21 SZ; 28 BPI; 7 OCD) | Caucasian Europeans (mostly Germans) |
|  | Franke et al, 2010 | 585 (HS) | European caucasian descent |

(Abbreviations, in alphabetical order): BD = Bipolar disorder; BDI = Bipolar disorder type I; HC = Healthy controls; HS = Healthy Subjects; OCD = Obsessive compulsive disorder; PH = Psychosis history; SZ = Schizophrenia; UR = Unaffected relatives.

**Table S22.** DISC1 and NRG1: primary studies of its SNPs of interest and their corresponding effects on brain volume; in comparison with the present study’s findings.

| **Primary study** | | | | | **Present study**  [113 (HC: 44; SZ: 14; BD: 21; ARMS: 34); White European & North American] | | | |
| --- | --- | --- | --- | --- | --- | --- | --- | --- |
| **Gene** | **SNP** | **Authors** | **Genotype effect^c^** | **Whole-brain region** | **Proxy SNP; allele (r^2^)** | **Statistical model^c^** | **Whole-Brain**  **Grey Matter (peak Z)** | **Whole-Brain**  **White Matter (peak Z)** |
| *DISC1* | rs11122319 | Kahler et al, 2012 | AA < AG < GG | Temporal lobe (Cortical thickness) | rs1417585; A~C, G~T (1.0) | CC *vs.* CT *vs.* TT | L Precuneus (Z=3.56, CC>TT) | n/a |
|  | rs2793092 | Mata et al, 2010 | AA > G-car.^a^; AA > AG > GG^b^ | Lateral Ventricles (total, R and L lateral) | rs2793098; A~A, G~G; (.99) | AA *vs.* G-car.; AA *vs.* AG *vs.* GG | R Mid Temporal (Z=4.11, AA>GG; AG>GG), Bilateral Thalamus (Z=3.86 and Z=3.81, AA<GG; AG<GG);  R Sup Frontal (Z=3.78, AG<GG; AA>AG); R Oper Portion Inf Frontal (Z=3.67, AG<GG; AA<GG) | R Supramarginal (Z=4.32, AA > G-car.);  R Mid Temporal (Z=4.00 AG>GG; AA>GG)  R Sup Frontal (Z=3.87, AG<GG) |
| *NRG1* | rs35753505 | Cannon et al, 2012 | (in SZ patients) C-car. < TT | R Uncinate fasciculus, R Inf Longitudinal fasciculus, R Ant limb of the internal capsule (WM) | N/A (Same SNP) | C-car. *vs.* TT^d^ |  |  |
|  |  |  | (in BDI patients) C-car. > TT | Cingulum, Parahippocampal gyrus, Callosal body (WM) |  |  |  |  |
|  | rs6994992 | Mata et al, 2009 | T-car. > CC | Lateral ventricles (total, R and L) | rs4733264; T~G, C~C; (.92) | G-car. *vs.* CC^d^ |  |  |
|  |  |  | TT > TC > CC | Lateral ventricles (total and left) |  | GG *vs.* GC *vs.* CC^d^ |  | n/a |
|  |  | McIntosh et al, 2008 | TT < CC | R Ant capsule (WM) |  |  |  |  |

Footnotes: originally reported as ^a^ TT vs. C-car. and ^b^ TT vs. TC vs. CC in Mata 2010; ^c^ risk genotype always to the left; ^d^ excluded due to “age” being found as true confounder variable.

(Abbreviations, in alphabetical order): Ant = Anterior; ARMS = At Risk Mental State; BD = Bipolar disorder; BDI = Bipolar disorder type I; car. = Carriers; HC = Healthy controls; Inf = Inferior; L = Left; Med = Medial; Mid = Middle; n/a = non-applicable; Oper = Opercular; Orb = Orbital; R = Right; Sup = Superior; SZ = Schizophrenia; Tri = Triangular; WM = White matter.

Coordinates of effects in tables S1 and S2.


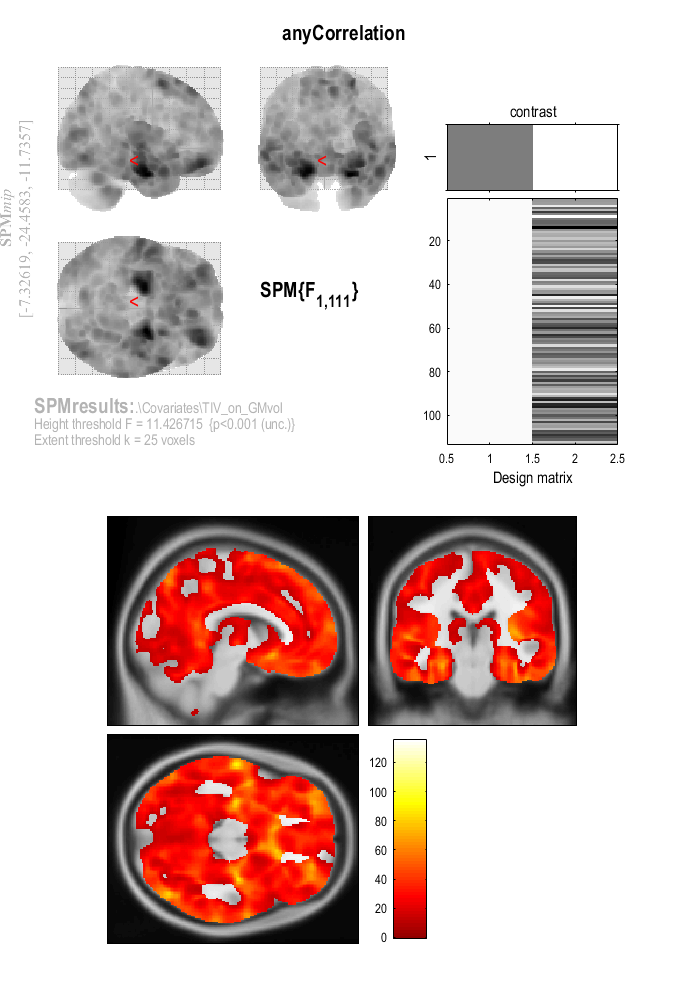

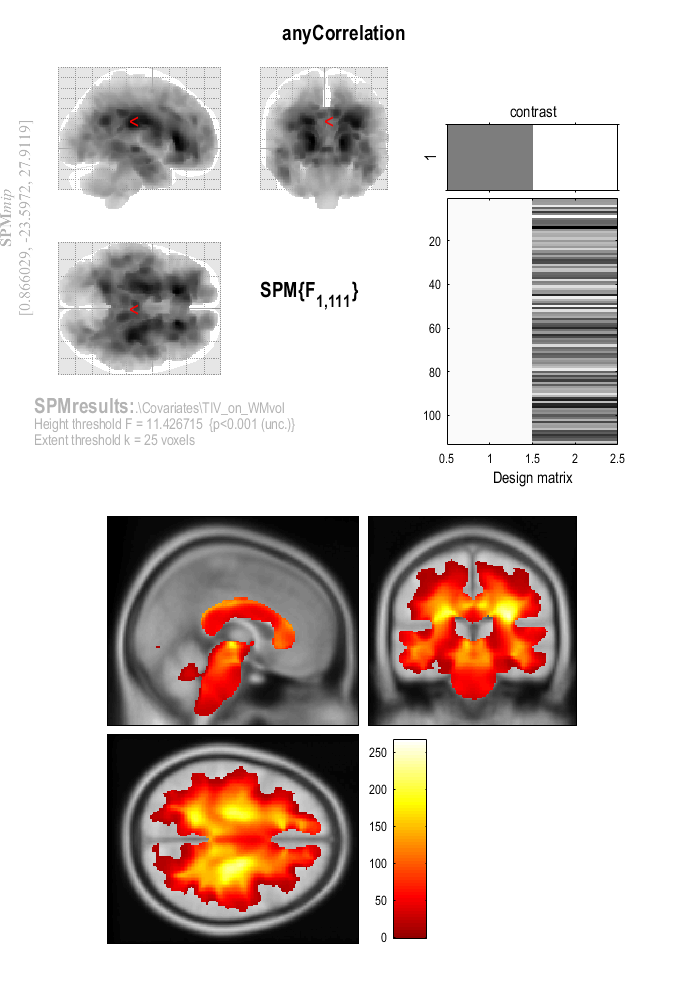


***Figure S1.*** *Effect of total intracranial volume on gray (left) and white (right) matter volumes.* Colorbar: 1-pvalue.


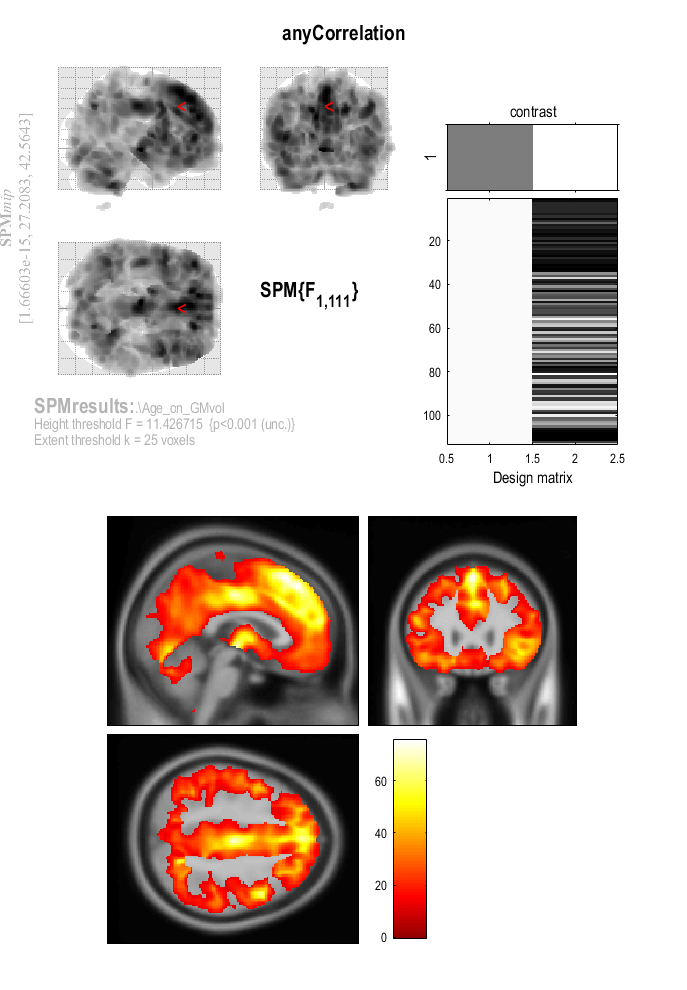

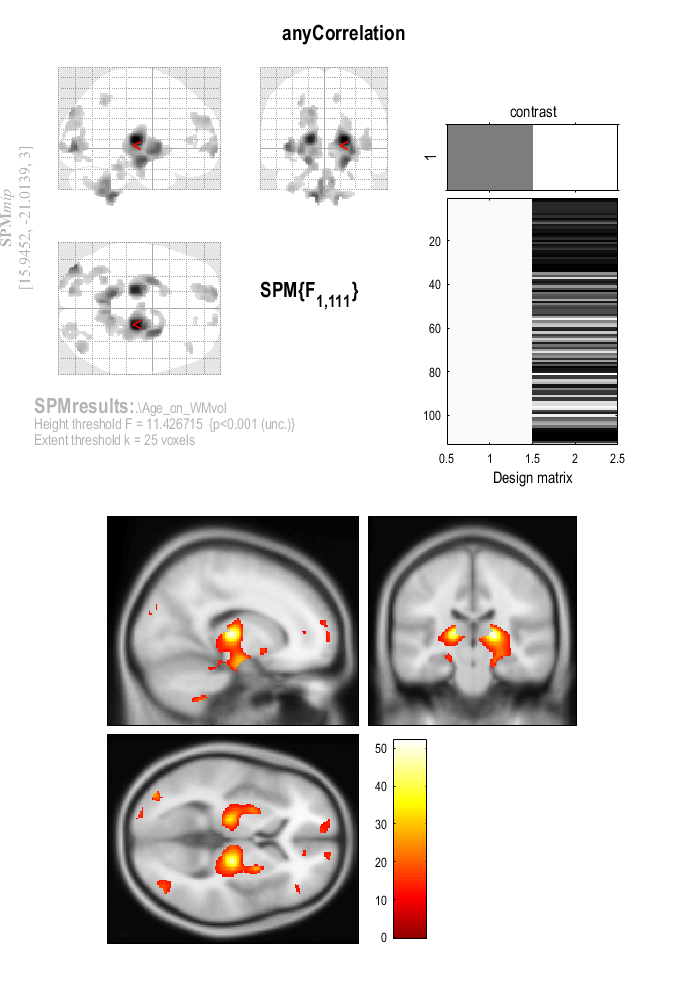


***Figure S2.*** *Effect of age at scan on grey (left) and white (right) matter volumes.* Colorbar: 1-pvalue.


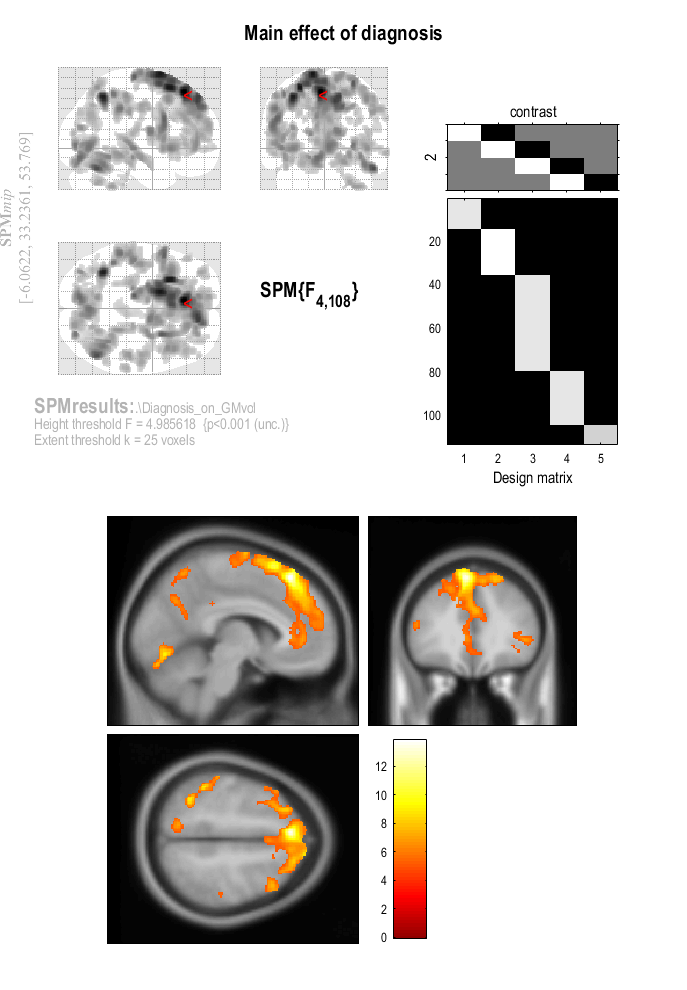

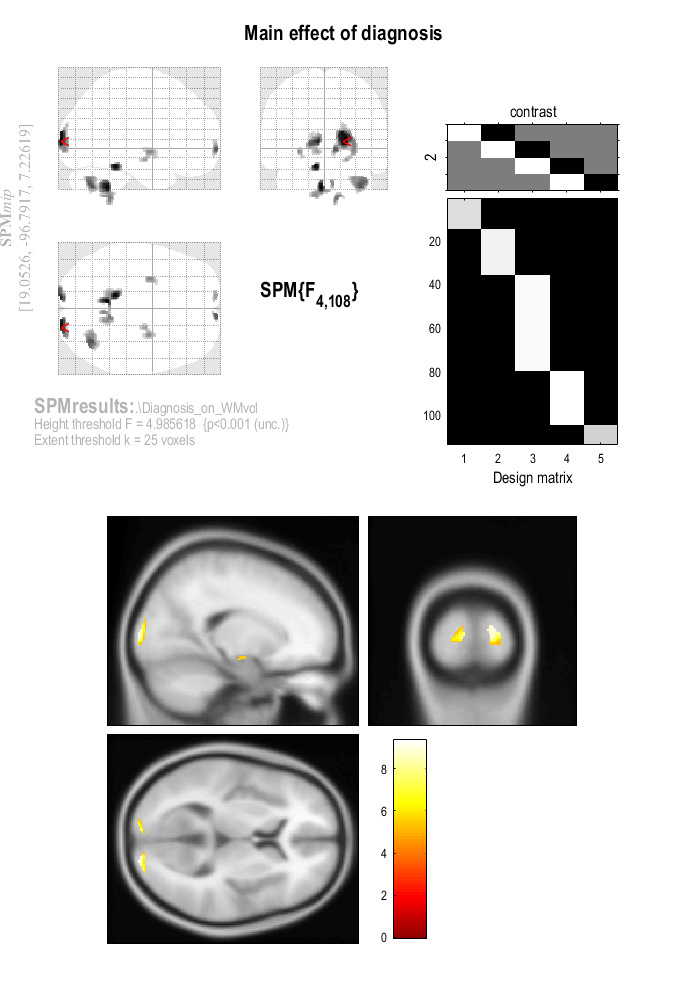


***Figure S3.*** *Effect of diagnosis on grey (left) and white (right) matter volumes.* Colorbar: 1-pvalue.


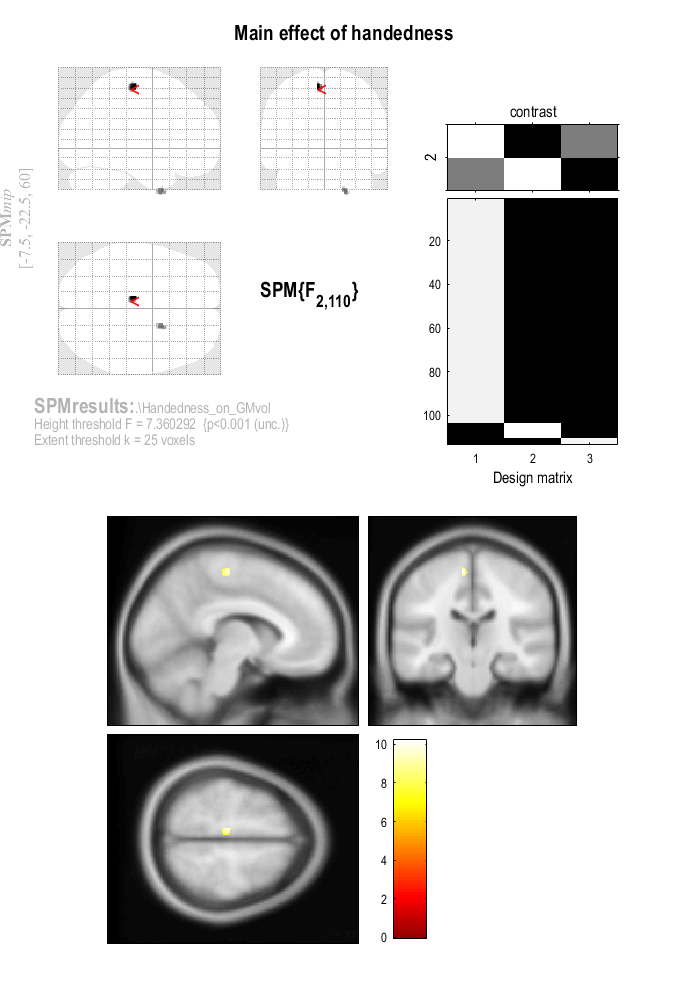

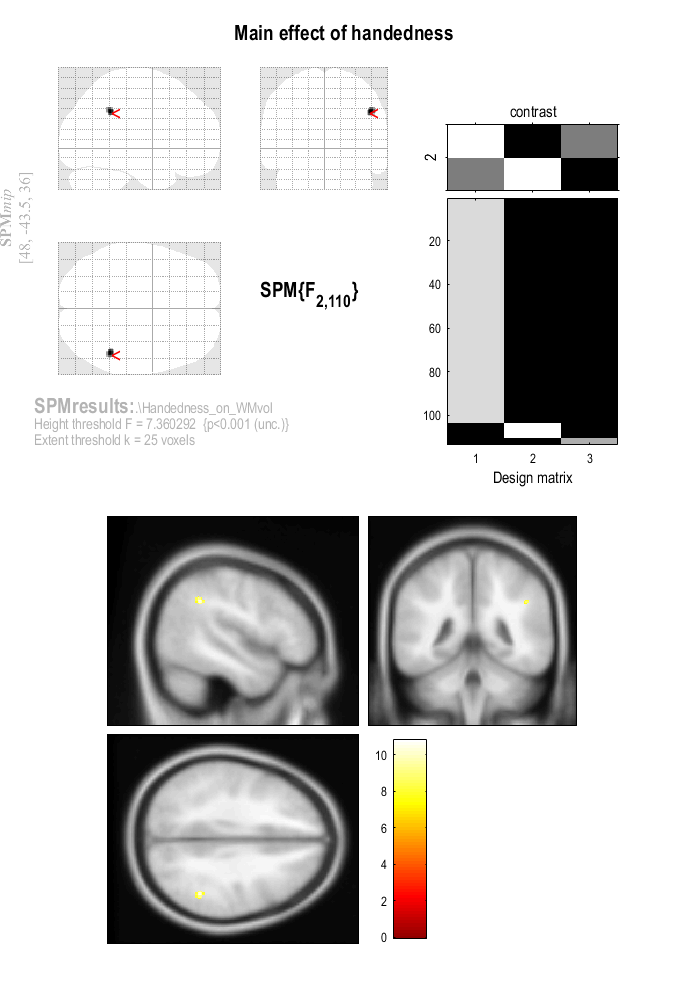


***Figure S4.*** *Effect of handedness on grey (left) and white (right) matter volumes.* Colorbar: 1-pvalue.


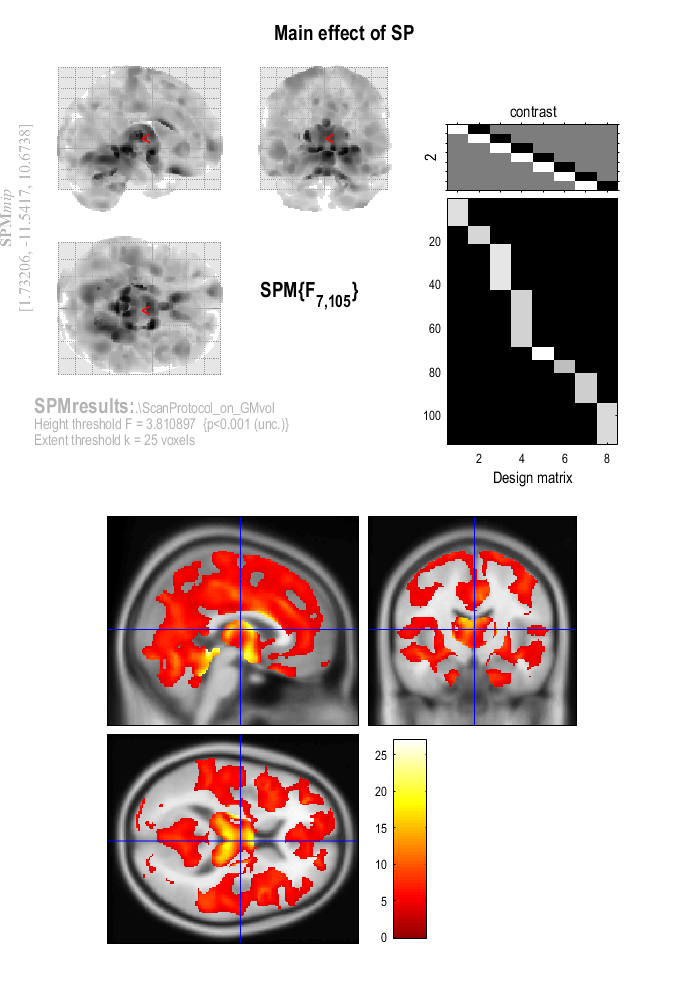

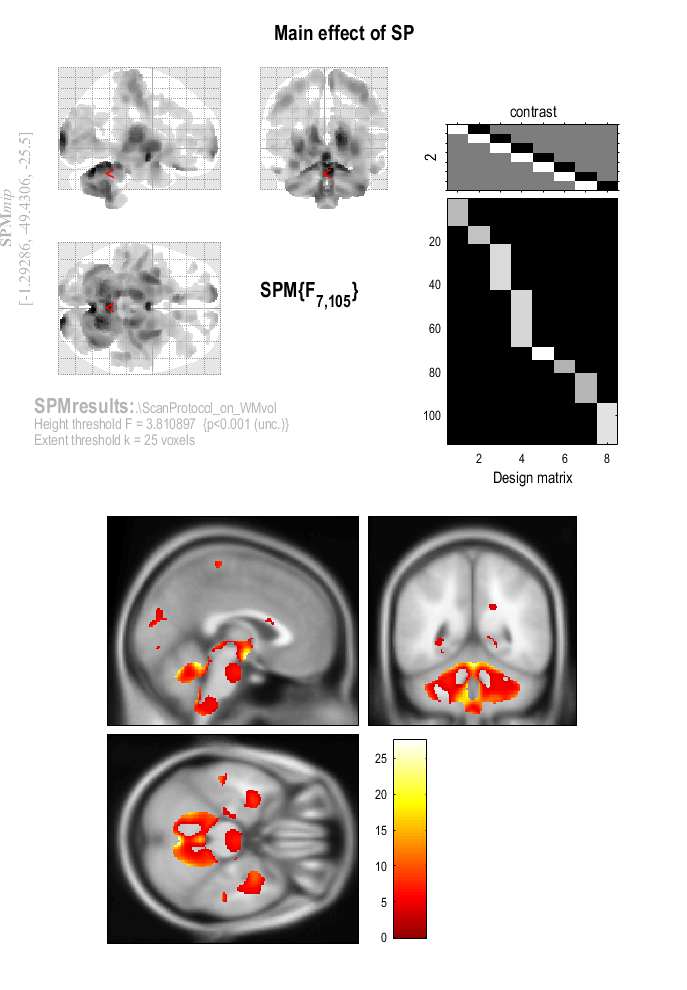


***Figure S5.*** *Effect of MRI scan protocol on grey (left) and white (right) matter volumes.* Colorbar: 1-pvalue.


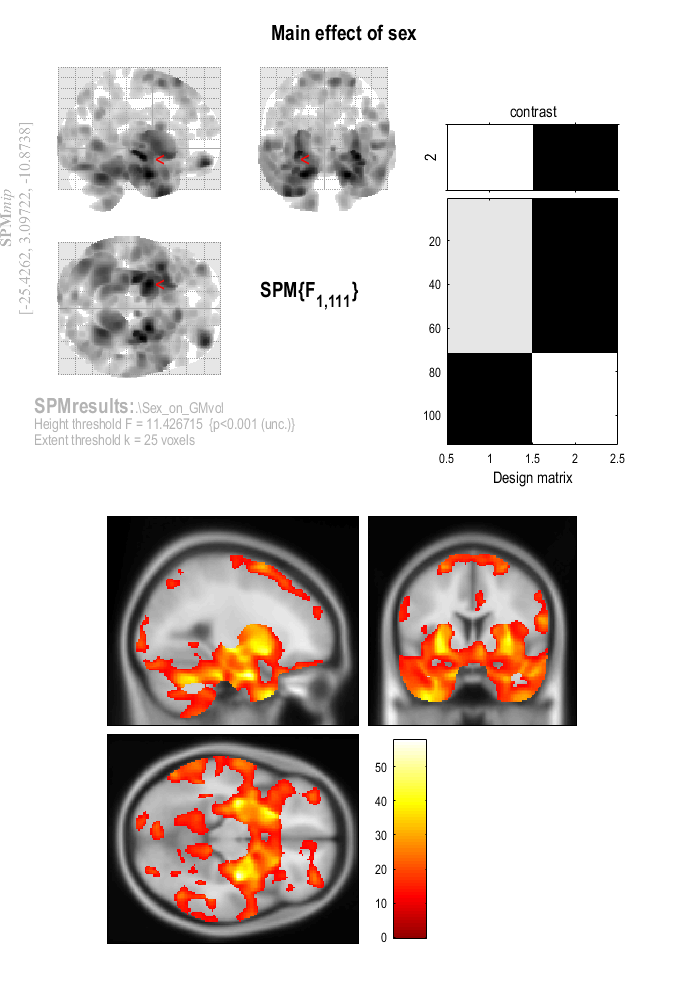

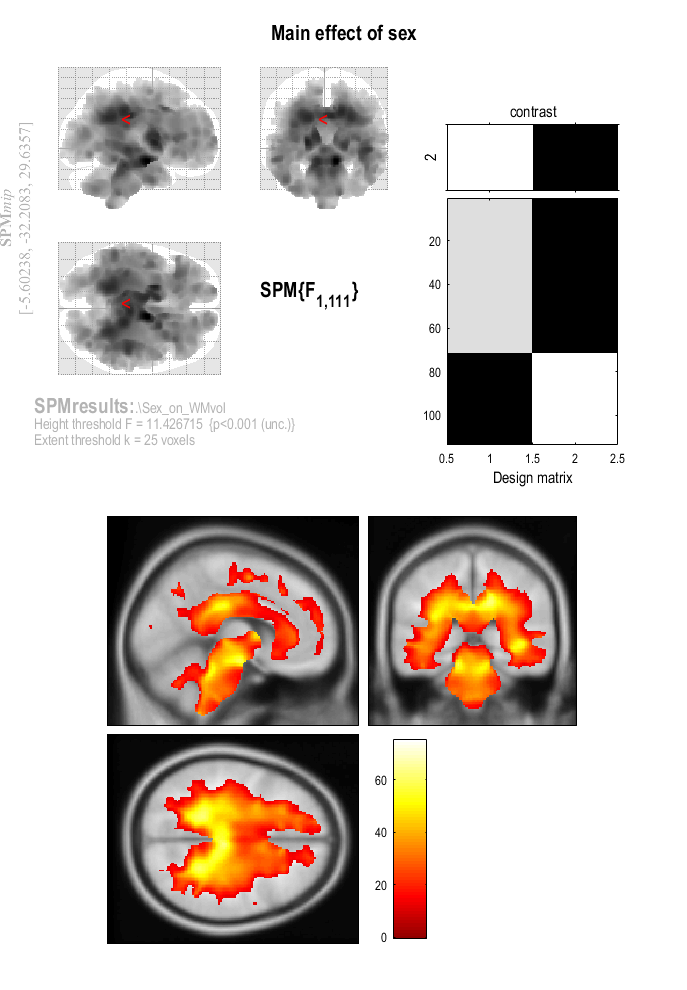


**Figure S6.** Effect of sex on grey (left) and white (right) matter volumes. Colorbar: 1-pvalue.

**
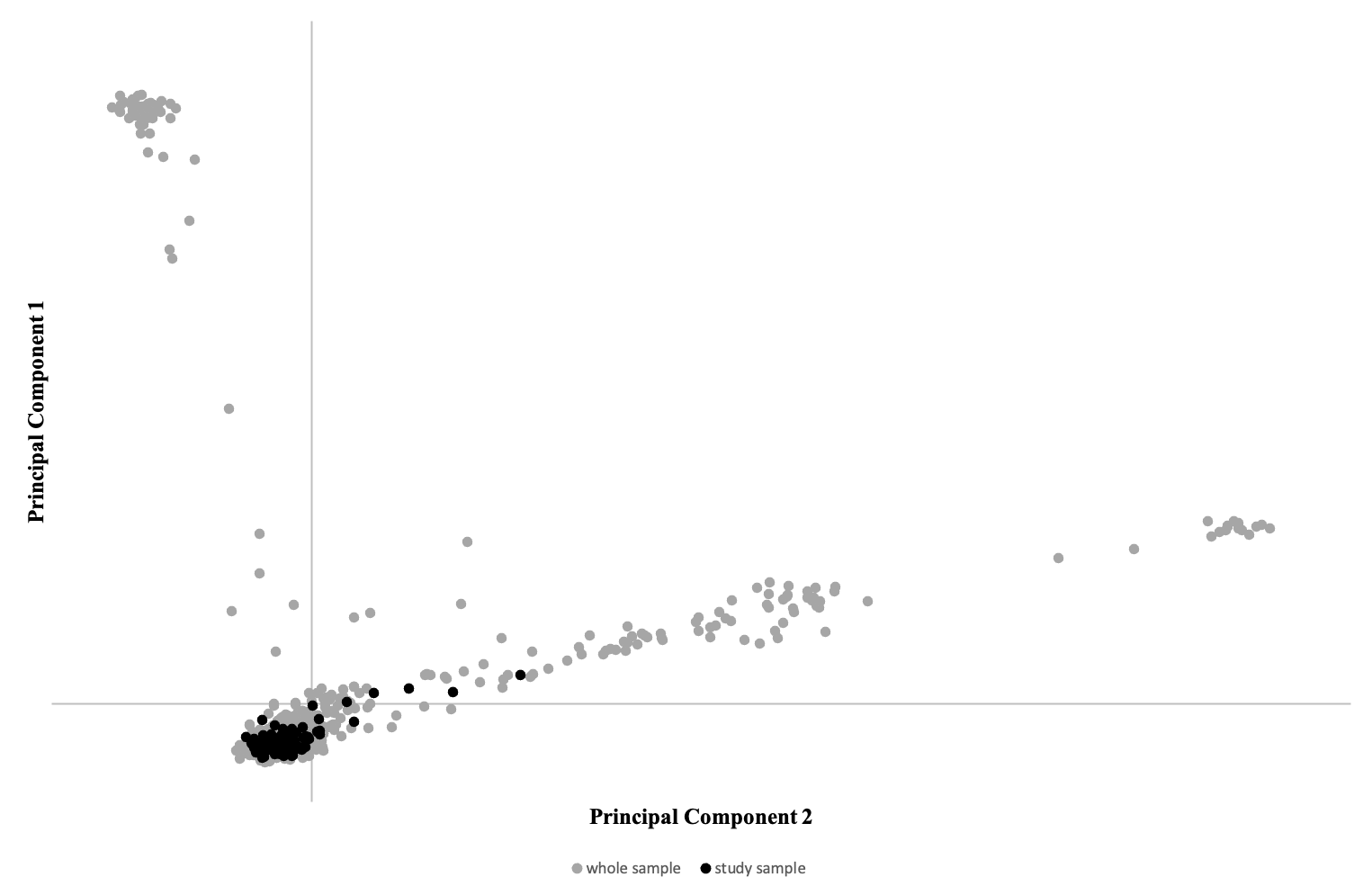

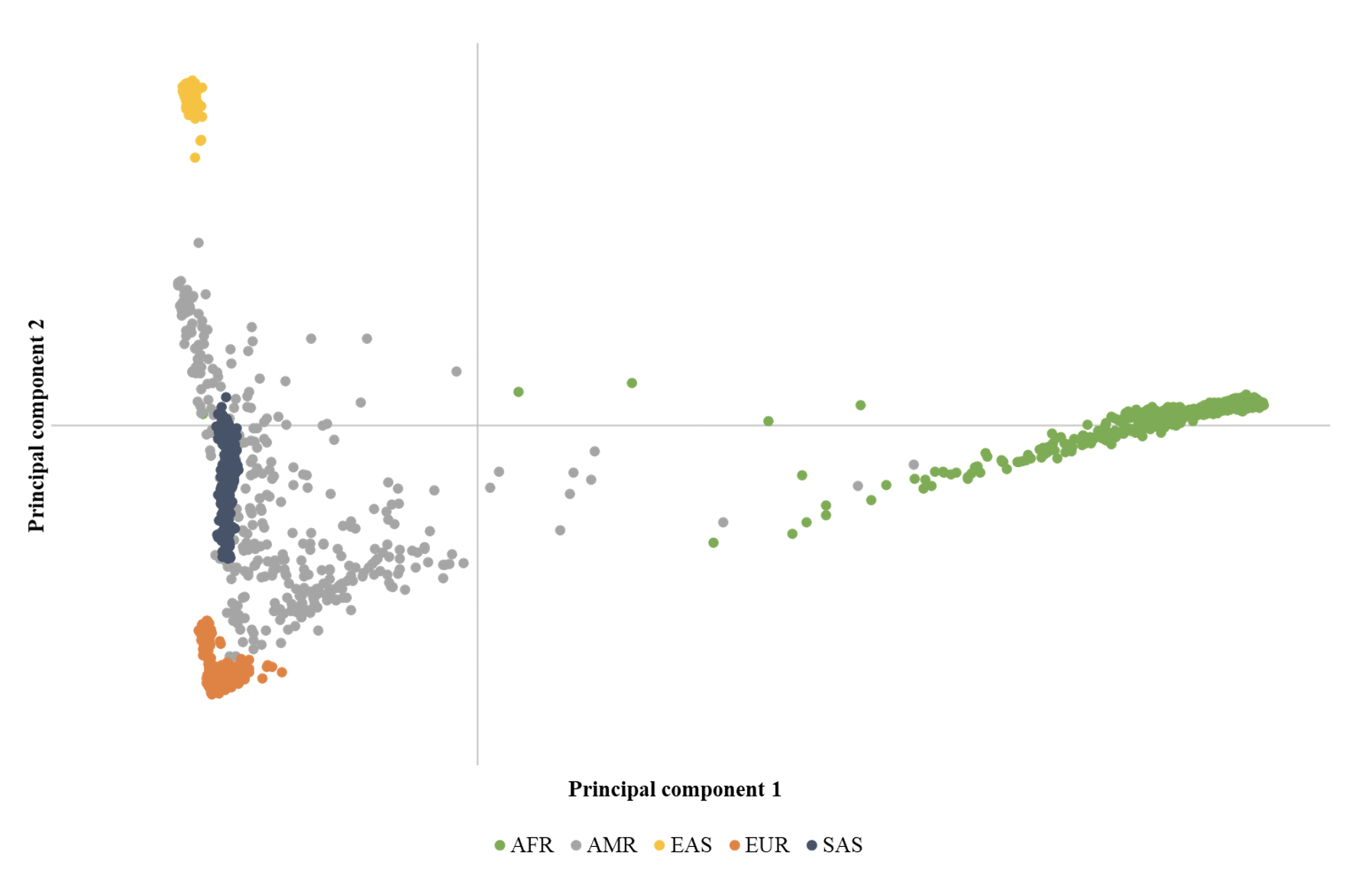
**

**Figure S7.** Population stratification analysis from the reference dataset (i.e. 1000 Genomes; top) and the study sample (i.e. the whole sample used for stratification analysis – gray – and the sample used in this study – black dots; bottom). Subjects selected for further analysis met the following two criteria: a) self-reported as being ‘white’ (black dots in the bottom plot); and b) show a genetic structure similar to the that of the reference dataset’s subjects with an European ancestry (orange dots in the top plot). All the subjects included in this study met the above two criteria. AFR: African, AS: Asian, AMR: American, EAS: East Asian, EUR: European, SAS: South Asian ancestries.

# References

1. Di Forti M, Marconi A, Carra E, et al. Proportion of patients in south London with first-episode psychosis attributable to use of high potency cannabis: A case-control study. *The Lancet Psychiatry*. 2015;2(3):233-238. doi:10.1016/S2215-0366(14)00117-5

2. American Psychiatric Association. *Diagnostic and Statistical Manual of Mental Disorders, Fourth Edition, Text Revision (DSM-IV-TR)*. Vol 1. Arlington, VA: American Psychiatric Association; 2000. doi:10.1176/appi.books.9780890423349

3. Prata DP, Mechelli A, Picchioni MM, et al. Altered effect of dopamine transporter 3′UTR VNTR genotype on prefrontal and striatal function in schizophrenia. *Archives of General Psychiatry*. 2009;66(11):1162-1172. doi:10.1001/archgenpsychiatry.2009.147

4. Broome MR, Woolley JB, Johns LC, et al. Outreach and support in south London (OASIS): implementation of a clinical service for prodromal psychosis and the at risk mental state. *European Psychiatry*. 2005;20(5):372-378. doi:10.1016/j.eurpsy.2005.03.001

5. Phillips LJ, Yung AR, McGorry PD. Identification of young people at risk of psychosis: validation of Personal Assessment and Crisis Evaluation Clinic intake criteria. *The Australian and New Zealand journal of psychiatry*. 2000;34 Suppl:S164-9. doi:10.1080/000486700239

6. Nurnberger  Jr JI, Blehar MC, Kaufmann CA, et al. Diagnostic Interview for Genetic Studies: Rationale, Unique Features, and Training. *Archives of General Psychiatry*. 1994;51(11):849-859. doi:10.1001/archpsyc.1994.03950110009002

7. Howie BN, Donnelly P, Marchini J. A Flexible and Accurate Genotype Imputation Method for the Next Generation of Genome-Wide Association Studies. *PLOS Genetics*. 2009;5(6):e1000529-. https://doi.org/10.1371/journal.pgen.1000529.

8. Marees AT, de Kluiver H, Stringer S, et al. A tutorial on conducting genome-wide association studies: Quality control and statistical analysis. *International Journal of Methods in Psychiatric Research*. 2018;27(2):e1608. doi:https://doi.org/10.1002/mpr.1608

9. Chang CC, Chow CC, Tellier LCAM, Vattikuti S, Purcell SM, Lee JJ. Second-generation PLINK: rising to the challenge of larger and richer datasets. *GigaScience*. 2015;4(1):s13742-015-0047-0048. doi:10.1186/s13742-015-0047-8

10. Gaser C, Dahnke R, Kurth K, Luders E. Alzheimer’s Disease Neuroimaging Initiative. A Computational Anatomy Toolbox for the Analysis of Structural MRI Data. *Neuroimage*. (in review).

11. Penny W., Friston K., Ashburner J., Kiebel S., Nichols T. *Statistical Parametric Mapping: The Analysis of Functional Brain Images*. 1st ed. London: Academic Press; 2006.

12. MATLAB. 2016.

13. [dataset] IXI dataset (RRID:SCR_005839).

14. Ashburner J. A fast diffeomorphic image registration algorithm. *NeuroImage*. 2007;38(1):95-113. doi:https://doi.org/10.1016/j.neuroimage.2007.07.007

15. Machiela MJ, Chanock SJ. LDlink: A web-based application for exploring population-specific haplotype structure and linking correlated alleles of possible functional variants. *Bioinformatics*. 2015;31(21):3555-3557. doi:10.1093/bioinformatics/btv402

16. Maldjian JA, Laurienti PJ, Kraft RA, Burdette JH. An automated method for neuroanatomic and cytoarchitectonic atlas-based interrogation of fMRI data sets. *NeuroImage*. 2003;19(3):1233-1239. doi:https://doi.org/10.1016/S1053-8119(03)00169-1

17. Prata DP, Mechelli A, Picchioni MM, et al. Altered effect of dopamine transporter 3′UTR VNTR genotype on prefrontal and striatal function in schizophrenia. *Archives of General Psychiatry*. 2009;66(11):1162-1172. doi:10.1001/archgenpsychiatry.2009.147

18. Tzourio-Mazoyer N, Landeau B, Papathanassiou D, et al. Automated anatomical labeling of activations in SPM using a macroscopic anatomical parcellation of the MNI MRI single-subject brain. *NeuroImage*. 2002;15(1):273-289. doi:10.1006/nimg.2001.0978
